# circRNA Profiling Reveals an Abundant circFUT10 that Promotes Adipocyte Proliferation and Inhibits Adipocyte Differentiation via Sponging let-7

**DOI:** 10.1016/j.omtn.2020.03.011

**Published:** 2020-03-30

**Authors:** Rui Jiang, Hui Li, Jiameng Yang, Xuemei Shen, Chengchuang Song, Zhaoxin Yang, Xiaogang Wang, Yongzhen Huang, Xianyong Lan, Chuzhao Lei, Hong Chen

**Affiliations:** 1 Shaanxi Key Laboratory of Animal Genetics, Breeding and Reproduction, College of Animal Science and Technology, Northwest A&F University, Yangling 712100, Shaanxi, China; 2 State Key Laboratory for Conservation and Utilization of Subtropical Agro-Bioresources, College of Animal Science and Technology, Guangxi University, Nanning 530004, China

**Keywords:** bovine, circRNA, adipocyte, let-7c

## Abstract

Adipose development is regulated by a series of complex processes, and non-coding RNAs (ncRNAs), including circular RNAs (circRNAs), play important roles in regulating proliferation and differentiation of adipocytes. In this study, we profiled circRNA expression in cattle fat tissue during calf and adult developmental stages and detected 14,274 circRNA candidates. Some circRNAs are differentially expressed between two developmental stages. We characterized *circFUT10*, named for its host gene *FUT10*, a highly expressed and abundant circRNA. Luciferase screening, an RNA-binding protein immunoprecipitation (RIP) assay, quantitative real-time PCR, and western blotting assays indicated that circFUT10 directly binds let-7c/let-e, and *PPARGC1B* (peroxisome proliferator-activated receptor γ coactivator 1-β) is identified as a target of let-7c. Flow cytometry, EdU (5-ethynyl-2′-deoxyuridine) incorporation, a CCK-8 (cell counting kit-8) assay, oil red O staining, and western blotting assays demonstrated that *circFUT10* promotes adipocyte proliferation and inhibits cell differentiation by sponging let-7c. The results demonstrate that *circFUT10* binding of let-7c promotes cell proliferation and inhibits cell differentiation by targeting *PPARGC1B* in cattle adipocytes.

## Graphical Abstract

**Figure:**
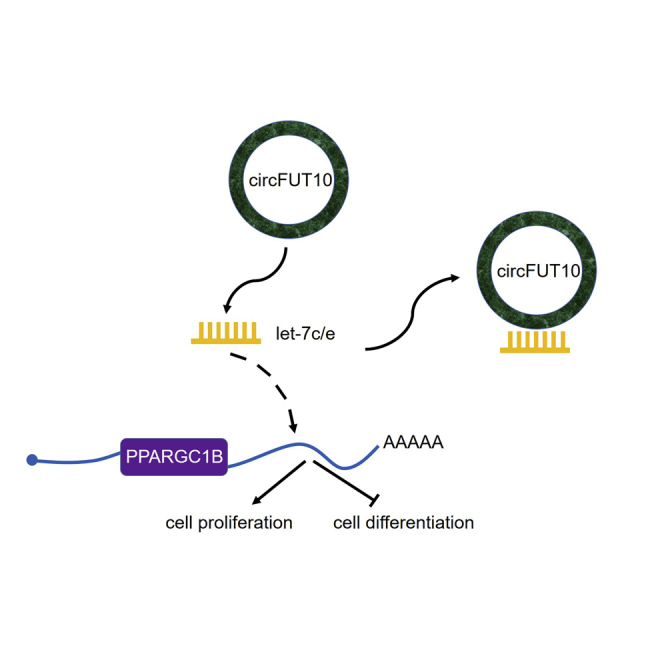

## Teaser

In order to verify the potential function of circRNA in adipose tissue, high-throughput sequencing of Qinchuan subcutaneous adipose tissue was performed, and circFUT10 was differentially expressed in calf and adult cattle adipose tissue. Experimental verification showed that circFUT10 promoted the proliferation of adipose cells and inhibited their differentiation by combining with let-7 family members.

## Introduction

Adipogenesis is a complex and precisely orchestrated process mediated by a network of adipocyte regulatory factors. The peroxisome proliferator-activated receptor γ (*PPARγ*) and CCAAT/enhancer-binding proteins (*C/EBPs*), a family of transcription factors composed of six members, named *C/EBPα* to *C/EBPζ*, mediate transcription.^1^^,^^2^ *PPARγ* is induced during terminal differentiation and is required for the activation of a number of genes with adipogenic.^3^ Three *C/EBPs* are involved in the differentiation of adipocytes *C/EBPα*, *C/EBPβ*, and *C/EBPδ*. *C/EBPβ* and *C/EBPδ* are expressed early in the induction of differentiation, and they initiate the transcription of *PPARγ* and *C/EBPα* to control the differentiation of adipocytes.^4^^,^^5^ Although the major molecular pathways of adipogenesis are understood, the regulatory scenario is far from complete. Recent studies have demonstrated roles of non-coding RNA (ncRNA) and circular RNA (circRNA) in the adipogenesis regulatory network.^6^^,^^7^

During the last decade, analysis of mammalian transcriptomes revealed that more than 50% of transcripts are not translated into proteins but are functional RNAs, including microRNAs (miRNAs), long ncRNAs (lncRNAs), and circRNAs. Although circRNAs were first found in RNA viruses as a viroid as early as 1976,^8^ and were later identified as endogenous RNA splicing products in eukaryotes in 1979,^9^ they were originally assumed to be the result of splicing error. In the 21st century, the development of RNA sequencing (RNA-seq) technologies and bioinformatics facilitated discovery of the abundance and diversity of circRNAs and allowed characterization of the dynamic expression patterns of circRNAs in various developmental stages and physiological conditions. Many circRNAs play integral roles in regulating gene activity at the transcriptional and post-transcriptional levels, by acting as scaffolds for the assembly of protein complexes, regulating alternative splicing or RNA-protein interactions, and functioning as miRNA sponges.^10^^,^^11^ Studies showed that circRNAs are abundant in eukaryotic cells, especially in mammalian brains,^12^ and some circRNAs have been associated with human neurodegenerative diseases.^13^ Interestingly, some circRNAs may encode micropeptides less than 100 aa in size that can perform specific micropeptide-mediated functions.^14^

Various ncRNAs have been identified in adipose tissues and can regulate adipogenesis. Several miRNAs significantly influence the physiology and pathology of adipose tissues, such as miR-14 and miR-143, which act in fat metabolism^15^^,^^16^ and can regulate adipocyte proliferation and differentiation *in vitro* and *in vivo*.^17^ lncRNAs are involved in transcriptional and epigenetic regulation by interacting with chromatin regulators, for example, acting as a “molecular scaffold” or decoy to activate or repress transcription and regulate adipogenesis. circRNAs have been identified and found to influence fat function in numerous species, including humans,^18^ mice,^19^ and pigs.^6^ *circFUT10*, *circFGFR4*, and *circLMO7* act as competing endogenous RNAs (ceRNAs) that bind miRNAs and regulate cell differentiation in cattle.^20^, ^21^, ^22^

Qinchuan cattle are a popular livestock breed, with a high muscle fat content and general stress resistance,^23^^,^^24^ and they were selected for this study. Our present study was intended to serve as a starting point for future studies examining the role played by circRNAs in bovine fat development. We characterized circRNA expression profiles in different developmental stages of bovine fat tissue. The Ribo-Zero RNA-seq method was used to compare whole transcriptomes of newborn calves and adult fat tissues. We then focused on *circFUT10*, which was found to be differentially and highly expressed in different developmental stages, and explored its role in cattle adipocyte proliferation and differentiation. The results indicated that *circFUT10* can function as a ceRNA for let-7c and can inhibit adipocyte differentiation, by altering the expression of its target gene *PPARGC1B* (*PPARγ* coactivator 1-β). This work serves as a valuable genomic resource for understanding the mechanisms of adipocyte differentiation and provides new insights into the identification and characterization of circRNA with functional roles in adipocyte proliferation and differentiation.

## Results

### Expression Profiles of circRNAs in Calf and Adult Bovine Fat Tissue

**Figure 1:**
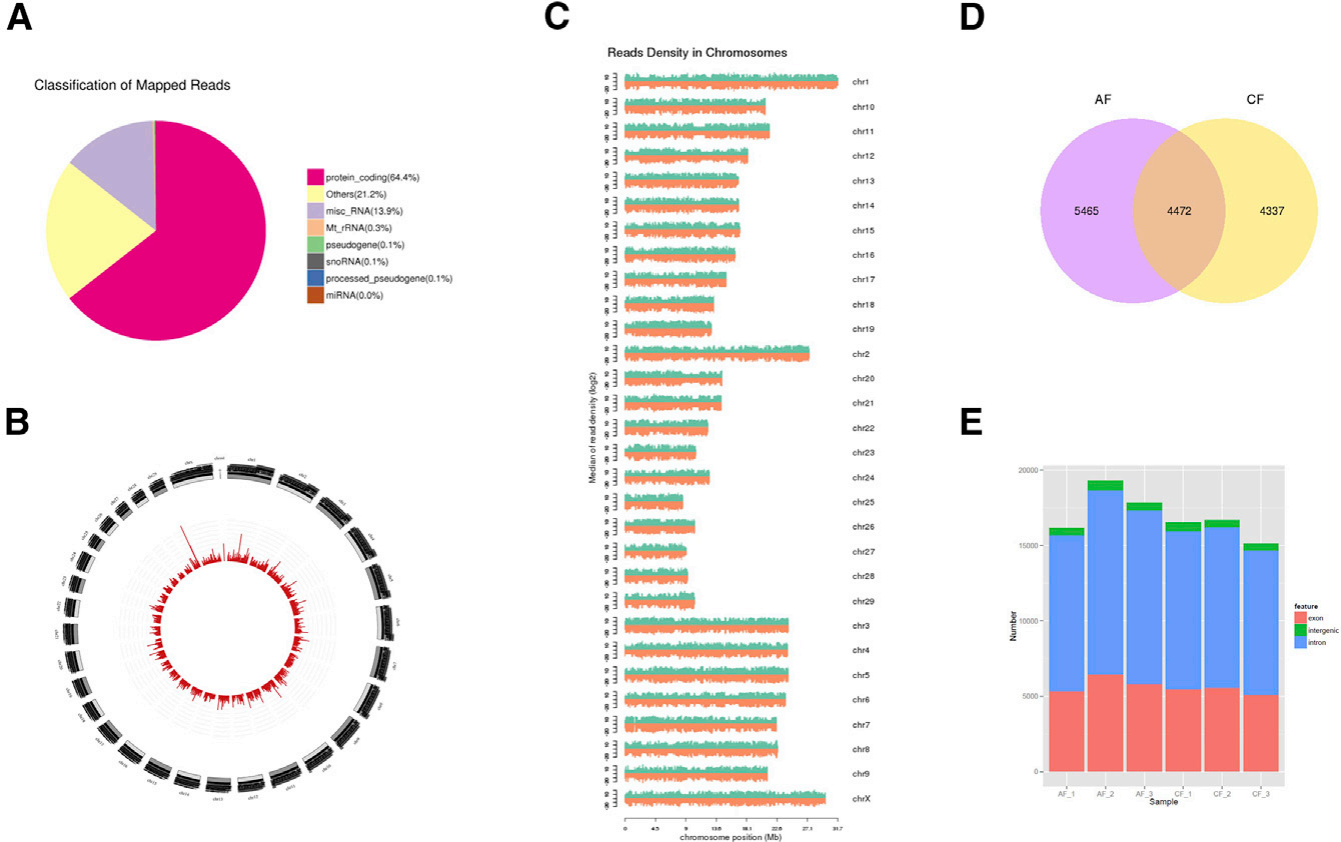
Identification of circRNAs in Bovine Fatty Tissue (A) The distribution of reads on the known gene types was obtained according to the expression distribution of each type of gene in the expression quantity statistics samples using HT-Seq software. (B) The total mapped reads were mapped to each chromosome. (C) The total mapped reads were mapped to the density of each chromosome on the genome. We use the ASCII code character sequence for chromosome sorting. (D) Venn diagram depicting different circRNAs uncovered at calf (CF) and adult cattle (AF) subcutaneous fatty tissue. 14,274 circRNA candidates were identified, with 4,337 and 5,465 specific to the calf and adult libraries, respectively. (E) Origin of circRNAs described in this study in the bovine genome.

We identified RNAs in calf and adult cattle fat tissue using Ribo-Zero RNA-seq,^25^ with 89–102 and 91–118 million unique mapped clean reads having been acquired from the calf and adult cattle libraries, respectively (Table 1). The expression distribution was determined for each gene based on the distribution of reads. We found that 64.4% of all of the reads mapped to the protein coding region, 13.9% of reads mapped to miscellaneous RNA, almost no reads mapped to pseudogene, small nucleolar RNA (snoRNA), or miRNA regions, and 21.2% of the reads were not mapped (Figure 1A). The total mapped reads were mapped to each chromosome (Figures 1B and 1C). The longer the length of the entire chromosome, the more the total mapped reads within the chromosome. The relationship between the number of reads on the localization chromosome and the number of reads can be more intuitive.

**Table 1:** Summary of Reads Mapping to the Cattle Reference Genome

Samples	Adult 1	Adult 2	Adult 3	Calf 1	Calf 2	Calf 3
Raw reads	94,367,280	119,126,430	109,773,454	105,005,806	91,779,236	93,247,554
Clean reads	90,859,922	117,571,292	105,144,278	101,605,688	88,865,576	89,537,724
Mapped reads	83,050,441	110,136,200	98,211,003	96,538,809	84,670,910	84,669,040
Mapping ratio	91.4%	93.68%	93.41%	95.01%	95.28%	94.56%
Uniquely mapped reads	69,609,751	100,240,434	89,608,961	88,815,771	77,779,872	77,656,770
Unique mapping ratio	76.61%	85.26%	85.22%	87.41%	87.53%	86.73%

We used “find-circ” to identify circRNAs^26^ in the libraries that were depleted of ribosomal RNA (rRNA) and identified 14,274 circRNA candidates. To comprehensively identify circRNAs with potential functions during calf and adult cattle fat tissue, the number of circRNAs in the two periods was counted. There were 4,337 circRNAs identified from the calf samples and 5,465 circRNAs identified from the adult fat tissue samples (Figure 1D). The identified circRNAs may include exon-derived circRNAs, intron-derived circRNAs, and circRNAs composed of exons and introns, so we next classified the sources of the sequenced circRNAs (Figure 1E).

### Differentially Expressed circRNAs in Bovine Fatty Tissues

**Figure 2:**
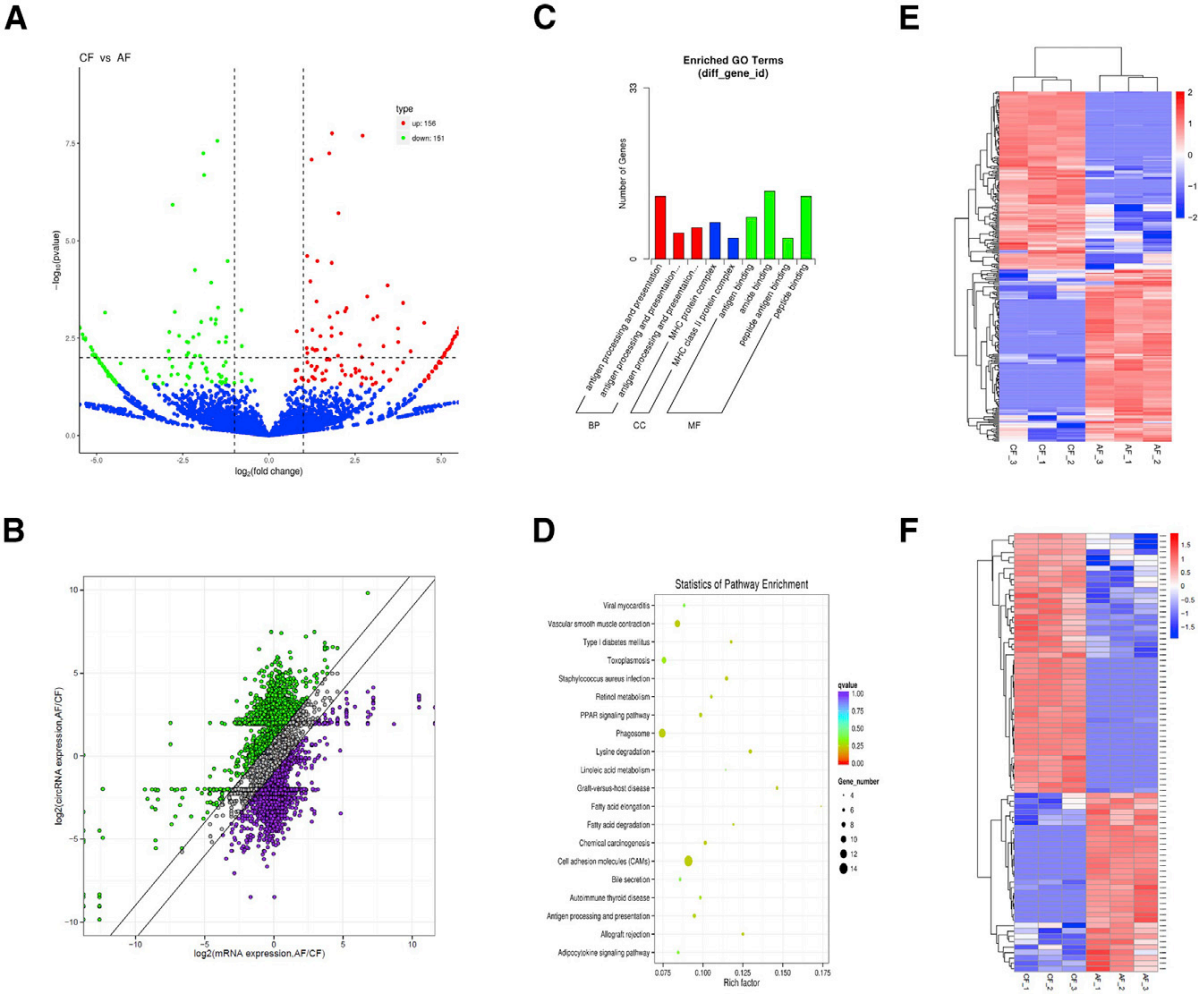
Differentially Expressed circRNAs in Bovine Fatty Tissues (A) Differential expression analysis using DESeq2. Volcano plot shows the correlation of abundances of individual circRNAs in adult or calf period. (B) Analysis of the impact of mRNA host gene expression change on circRNA expression change in fatty tissue. (C and D) GO (C) and KEGG (D) terms of significantly differentially expressed circRNAs (p < 0.05). (E) Clustered heatmap of differentially expressed circRNAs (p < 0.05). (F) Clustered heatmap showing abundances of the corresponding linear host transcripts of the 100 most differentially expressed circRNAs (p < 0.05).

Based on the fold change and the adjusted level of significance, differential circRNAs of adult cattle and calves were identified, with 151 circRNAs downregulated and 156 circRNAs upregulated when comparing calf tissue to adult tissue (p < 0.05, Figure 2A). To determine whether the expression of circRNA correlated with mRNA changes for different development stages, we directly compared changes in mRNA and circRNA expression between calf and adult fat tissues. We found that the expression pattern of many circRNAs was different in different developmental stages, but that altered mRNA expression cannot sufficiently explain the observed differences in circRNA expression, suggesting that circRNAs are not byproducts of inaccurate canonical splicing (Figure 2B).

GO (Gene Ontology) and KEGG (Kyoto Encyclopedia of Genes and Genomes) analysis were used to uncover the role of differentially expressed circRNAs and determine the signal transduction pathways involved in the host genes of circRNAs (Figures 2C and 2D). The GO and KEGG terms of host genes encoding differentially expressed circRNAs (p < 0.05) are displayed in Table S1. To further explore the potential functions of circRNAs, we prepared a clustered heatmap of differentially expressed circRNAs (p < 0.05, Figure 2E) that includes the abundances of the corresponding linear host transcripts of the 100 most differentially expressed circRNAs (Figure 2F). Interestingly, the expression of the most differentially expressed circRNA is consistent with the expression of its host gene, but the circRNA accounts for a relatively high expression level compared to its host gene.

### Characteristics of circRNA in Bovine Fatty Tissues

In order to understand the potential functions of circRNAs, we analyzed the characteristics of circRNAs in fatty tissue. We found that most circRNAs consist of one to four exons in calves or adult cattle (Figure S1A). One host gene can produce 1–5 circRNAs, and even some genes produce more than 10 circRNAs (Figure S1B). The genomic distance of the back-splicing site in most circRNAs is within 50 kb, with only a few circRNAs spanning >100 kb, suggesting that circRNAs are likely generated within the same gene region and may arise from RNA splicing throughout development and growth of adipocytes (Figure S1C). The exon length of most circRNAs is less than 5,000 nt, and the length of a single-exon circRNA is much greater than the exon length of a multi-exon circRNA (Figures S1D–S1F).

### ceRNA Network in Bovine Fatty Tissues

Some circRNAs have a miRNA binding site, allowing them to competitively bind to miRNA as a miRNA sponge, inhibiting the ability of miRNAs to bind their target genes and thereby indirectly regulating gene expression. This phenomenon suggests that circRNAs could act as ceRNAs, and miRNAs can be targeted by both circRNAs and mRNAs. To construct a ceRNA network, we selected four circRNAs whose host genes were associated with fat development and predicted their binding miRNAs from our high-throughput sequencing data. Based on the presence of potential binding sites in circRNAs, we constructed a miRNA-circRNA network (Figure S2A).

### The Expression of circFUT10 in Calf and Adult Cattle Adipose Tissue

**Figure 3:**
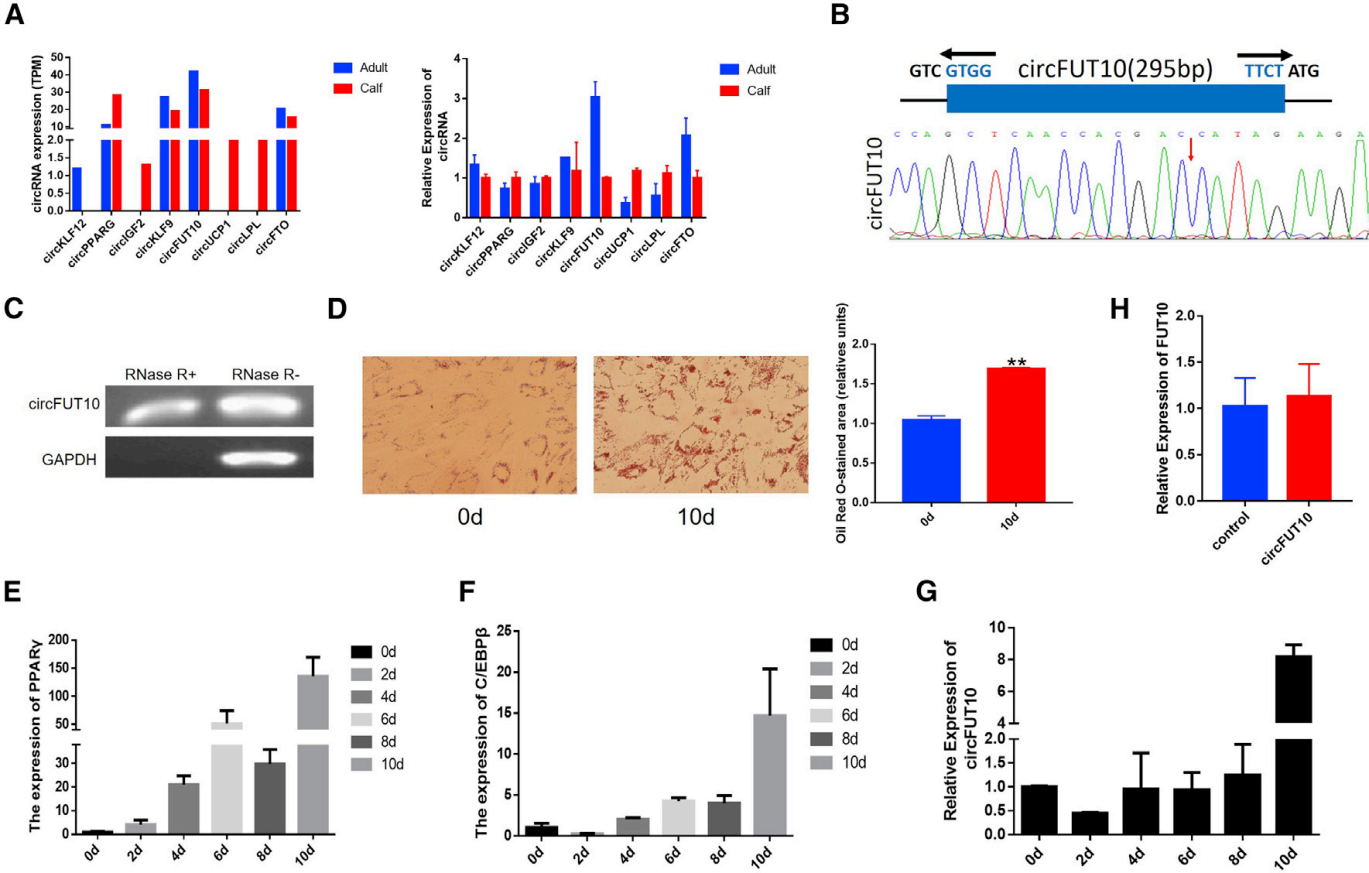
Expression of *circFUT10* in Calf and Adult Cattle Adipose Tissue (A) The expression of eight circRNAs whose maternal genes were associated with fat development in our sequencing results. Validation of the expression of these eight circRNAs in three calf and adult fat tissues using qPCR is shown. (B) Schematic view illustrating the design of primers for circFUT10 used in qPCR and Sanger sequencing of the junction of back-spliced products. (C) *circFUT10* exhibits obvious resistance to RNase R digestion. (D–F) Oil Red O staining (D) and qPCR analysis of adipogenesis markers *PPARγ* (E) and *C/EBPβ* (F) confirm the identity of bovine preadipocytes; quantitative analysis of oil Red O staining was performed using ImageJ. (G) The expression dynamics of *circFUT10* during adipocyte differentiation were determined by qPCR, the expression of *circFUT10* was normalized to 1 at day 0 of differentiation, and the expression of other days of differentiation was corrected according to this standard. (H) The expression level of *FUT10* gene was measured when *circFUT10* was overexpressed in bovine fat cells. Values are mean ± SEM for three biological replicates. ∗p < 0.05, ∗∗p < 0.01.

To verify the abundance of circRNAs obtained by RNA-seq, we randomly selected eight circRNAs with host genes that are associated with fat development and amplified their junction regions using specific qPCR primers. The results showed that the expression levels of these circRNAs in calves and adult cattle were similar to those indicated by the circRNA-seq data, supporting the rationality of the sequencing data. The highest expression level of the eight tested circRNAs was observed for *circFUT10*, suggesting that it may play a role in fat development (Figure 3A). We confirmed the size and sequence of the amplified PCR product with specific circRNA junctions by Sanger sequencing (Figure 3B). An RNase R digestion assay was performed and further demonstrated that *circFUT10* is circular in form (Figure 3C). To determine the effect of *circFUT10* on cell differentiation, bovine adipocytes were induced to differentiate for 10 days and then analyzed with oil Red O staining (Figure 3D). As expected, the expression levels of established adipocyte markers, peroxisome proliferator-activated receptor γ (*PPARγ*) and CCAAT/enhancer-binding protein β (*C/EBPβ*), were significantly increased (Figures 3E and 3F). More importantly, in the isolated bovine adipocytes, *circFUT10* was more highly expressed in the cell differentiation stage than in the proliferation phase, revealing a potential role of *circFUT10* in adipocyte differentiation (Figure 3G). There was no significant change in the level of the host gene *FUT10* after overexpression of *circFUT10*, indicating that the effect of *circFUT10* on adipocytes is not related to the level of its host gene (Figure 3H). Therefore, *circFUT10* was chosen as a candidate circRNA that is transcribed from the *FUT10* gene in chromosome 27.

### circFUT10 Promotes Adipocyte Proliferation

**Figure 4:**
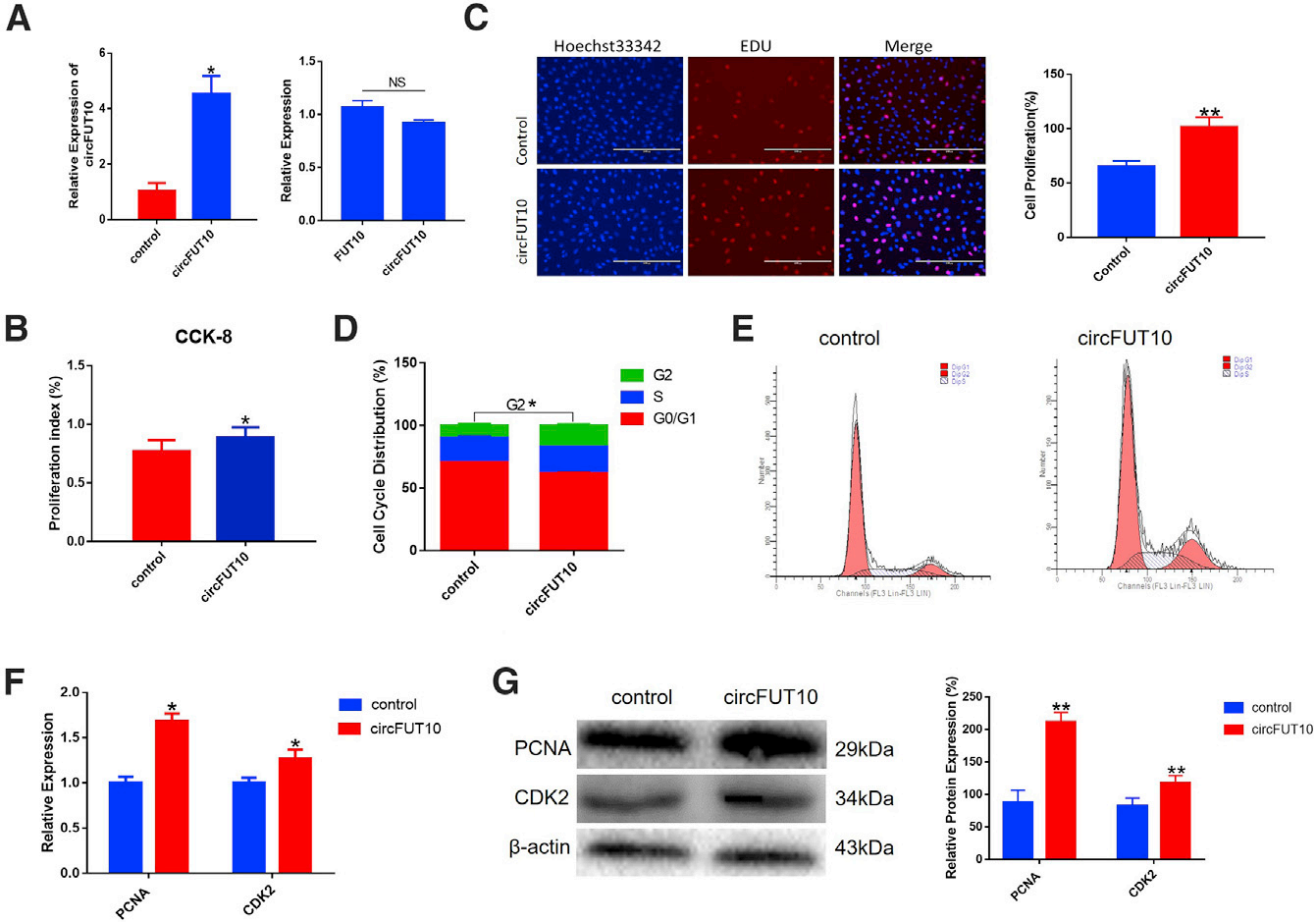
Effect of *circFUT10* on the cell proliferation (A) Visualization of the efficiency of *circFUT10* overexpression vector pCD2.1-*circFUT10* by qPCR. (B and C) Cell proliferation analysis using a Cell Counting Kit-8 (CKK-8) assay (B) and EdU incorporation assays (C). Scale bars represent 100 mm. (D and E) Bovine primary fat cells were transfected with pCD2.1-circFUT10, cell phases were analyzed by flow cytometry (E) and made statistics (D). (F and G) The expression of proliferating cell nuclear antigen (*PCNA*) and cyclin-dependent-kinase 2 (*CDK2*) were detected by qPCR (F) and western blot (G); quantitative analysis of western blot was performed using ImageJ. Values are mean ± SEM for three biological replicates. ∗p < 0.05, ∗∗p < 0.01.

The *circFUT10* expression plasmid pCD2.1-*circFUT10* was transfected into bovine adipocytes and significantly enhanced *circFUT10* expression level. After overexpression of *circFUT10*, the expression of the *FUT10* gene was not significantly different from that of *circFUT10*, indicating that pCD2.1-*circFUT10* has a high efficiency of circularization (Figure 4A). The CCK-8 (Cell Counting Kit-8) assay demonstrated that adipocytes in which the *circFUT10* was significantly overexpressed significantly promoted cell proliferation compared to control cells (Figure 4B). Similarly, the EdU (5-ethynyl-2′-deoxyuridine) staining assay showed that the increased expression of *circFUT10* increased the number of EdU-positive cells, suggesting a proliferative effect of *circFUT10* (Figure 4C). Cell cycle analysis revealed that *circFUT10* increased the number of adipocytes in the S and G_2_ phases, and decreased the proportion of cells in the G_0_/G_1_ phase (Figures 4D and 4E). Consistently, pCD2.1-*circFUT10* increased the expression of cyclin-dependent kinase 2 (*CDK2*) and proliferating cell nuclear antigen (*PCNA*) at both the mRNA and protein levels (Figures 4F and 4G), and knockdown of circFUT10 significantly decreased the expression of *CDK2*, *cyclin D1*, and *PCNA* (Figure S3). These results confirm that *circFUT10* promotes cell proliferation in bovine adipocytes.

### circFUT10 Inhibits Adipocyte Differentiation

**Figure 5:**
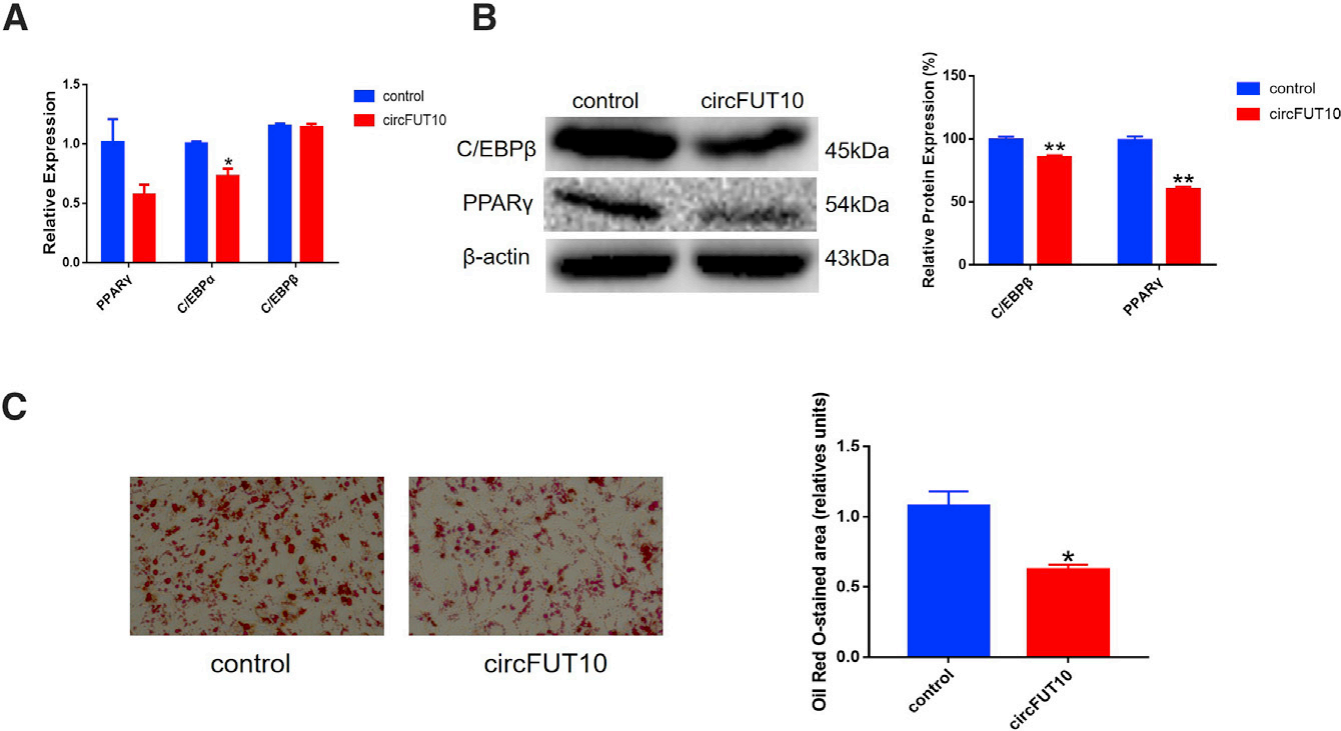
*circFUT10* Inhibits Differentiation of Bovine Adipocytes (A) Expression levels of *PPARγ*, *C/EBPα*, and *C/EBPβ* in bovine adipocytes by qPCR. (B) Expression levels of *PPARγ* and *C/EBPβ* in bovine adipocyte by western blot. Quantitative analysis of western blot was performed using ImageJ. The expression levels of the marker genes in the control group were normalized to 1 or 100%, and the expression levels of the marker genes in the overexpressed *circFUT10* group were normalized according to this standard. (C) Overexpression of *circFUT10* inhibited adipogenesis, as indicated by Oil Red O staining, and the quantitative analysis of oil Red O was performed using ImageJ. ∗p < 0.05, ∗∗p < 0.01.

To assess the effect of *circFUT10* on adipocyte differentiation, the expression levels of *PPARγ* and *C/EBPs* were detected in primary cattle adipocytes treated with pCD2.1-*circFUT10*. Next, qPCR was used to detect the mRNA expressions levels of *PPARγ*, *C/EBPα*, and *C/EBPβ*. We found that *circFUT10* overexpression significantly inhibited the expression of PPARγ and *C/EBPα* (Figure 5A). Similarly, western blot analysis revealed that *circFUT10* inhibited the expression of both *PPARγ* and *C/EBPβ* (Figure 5B). The oil red O staining assay results showed significantly fewer lipid droplets in the *circFUT10*-overexpressing group compared to the number in the control group (p < 0.05; Figure 5C). After knockdown of *circFUT10*, the expression of *CEBP/β* was significantly higher than the control group in mRNA and protein levels (Figure S4). These results confirmed that *circFUT10* plays an inhibitory role in the process of fat differentiation, which is not conducive to fat deposition.

### circFUT10 Acts as a ceRNA for let-7c

**Figure 6:**
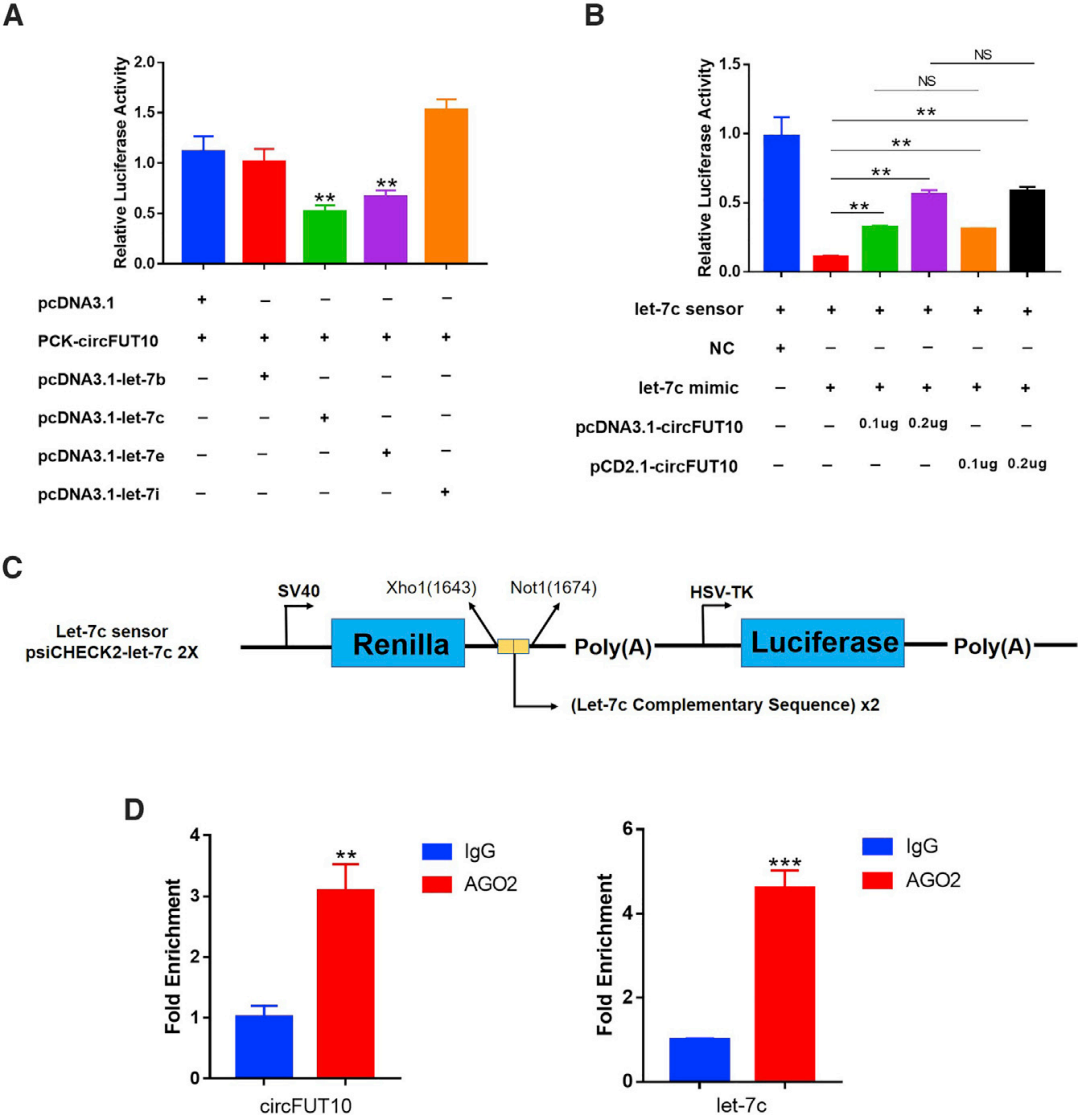
*circFUT10* Acts as a ceRNA for let-7c (A) let-7b, let-7c, let-7e, and let-7i were co-transfected with pCK-*circFUT10* into HEK293T cells (eight replicates in each group, each replicate adding 0.1 μg of miRNA plasmid and 0.1 μg of pCK-*circFUT10* plasmid). Renilla luciferase activity was normalized to firefly luciferase activity. (B) let-7c sensor construct and co-transfected with let-7c mimic or pcDNA3.1-*circFUT10* into HEK293T cells. (C) Schematic diagram of the let-7c sensor structure. (D) Association of *circFUT10* and let-7c with Ago2. *circFUT10* and let-7c were transfected into adipocytes for 24 h and lysed; cellular lysates were used for the RIP assay with Ago2 antibody. *circFUT10* and let-7c levels were detected using qPCR. ∗p < 0.05, ∗∗p < 0.01.

It has been reported that circRNA may regulate gene expression at the post-transcriptional level.^21^ The online software RNAhybrid (https://bibiserv.cebitec.uni-bielefeld.de/rnahybrid) was used to predict the miRNAs binding sites of *circFUT10*. We found a potential binding site for *circFUT10* to bind to the seed region of the let-7 family (Table S2), consistent with the sequencing data (Figure S2A). We next selected the first four let-7 family members with the lowest binding energy, including let-7b, let-7c, let-7e, and let-7i. Luciferase assay revealed that let-7c significantly inhibited Renilla luciferase (Rluc) expression of pCK-*circFUT10* (Figure 6A). Additionally, the sensor analysis further verified the direct binding between *circFUT10* and let-7c (Figures 6B and 6C). Next, an RNA-binding protein immunoprecipitation (RIP) assay was performed using Ago2 antibody, followed by qPCR, and the results confirmed that both *circFUT10* and let-7c can interact with the AGO2 protein, indicating a potential interaction between *circFUT10* and let-7c. (Figure 6D). Members of the let-7 family play important roles in metabolic diseases.^27^ We overexpressed let-7c in fat cells, and the expression levels of *IRS1*, *TGFBR1*, and *TGFBR2* genes changed significantly at the mRNA level. This effect was reversed after co-transformation of *circFUT10* (Figure S2B).

**Figure 7:**
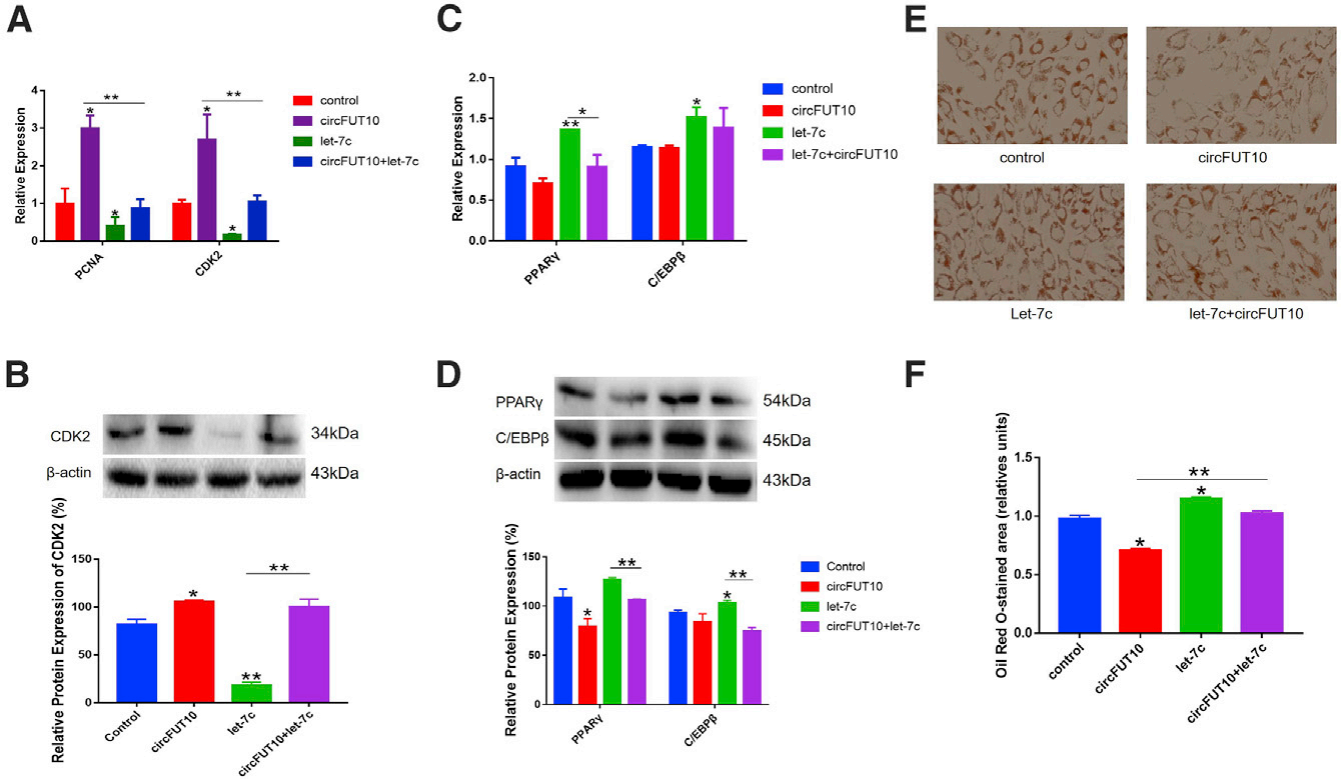
*circFUT10* Binding of let-7c Promotes Adipocyte Proliferation and Inhibits Its Differentiation (A and B) Fat cells were co-transfected with the let-7c mimic and *circFUT10*; the expression levels of proliferating cell nuclear antigen (*PCNA*) and cyclin-dependent-kinase 2 (*CDK2*) were detected by qPCR (A) and western blot (B). (C and D) The expression levels of *PPARγ* and *C/EBPβ* in bovine adipocytes were measured by qPCR (C) and western blot (D). (E and F) Oil red O staining was detected after fat cells were co-transfected with the let-7c mimic and circFUT10 (E). Quantitative analysis of western blot and oil red O were performed using ImageJ (F). Control, 1 μg of pCD2.1 plasmid + 2.5 μL of mimic NC; *circFUT10*, 1 μg of *circFUT10* plasmid + 2.5 μL of mimic NC; let-7c, 1 μg of pCD2.1 plasmid + 2.5 μL of let-7c mimic; *circFUT10*+let-7c, 1 μg of *circFUT10* plasmid + 2.5 μL of let-7c mimic. ∗p < 0.05, ∗∗p < 0.01.

To determine whether the regulatory effect of *circFUT10* on cell proliferation and differentiation was dependent on the interaction with let-7c, let-7c mimics or/and pCD2.1-*circFUT10* were transfected into bovine adipocytes. We found that let-7c decreased the expression of *CDK2* and *PCNA* at the mRNA and protein levels, but these effects were countered by overexpression of circFUT10. The expression of marker genes *PPARγ* and *CEBP/β* were significantly higher in the differentiation period than the control group with let-7c overexpression and showed a downward trend after co-transfection with *circFUT10* (Figures 7A–7D). The oil red O staining assay showed that the lipid droplets in the overexpressing let-7c group were significantly higher than those in the control group, but these effects were improved by overexpression of *circFUT10* (Figures 7E and 7F). let-7e also significantly inhibited Rluc expression of pCK-*circFUT10* (Figure 6A), suggesting that it binds to *circFUT10* and contributed to the proliferation and differentiation of adipocytes. Results of qPCR and western blot indicated that let-7e can indeed inhibit the proliferation of adipocytes and promote their differentiation, and it picked up after co-transfection of *circFUT10* (Figures S2C and S2D).

### *PPARGC1B* Is a Target of let-7c

**Figure 8:**
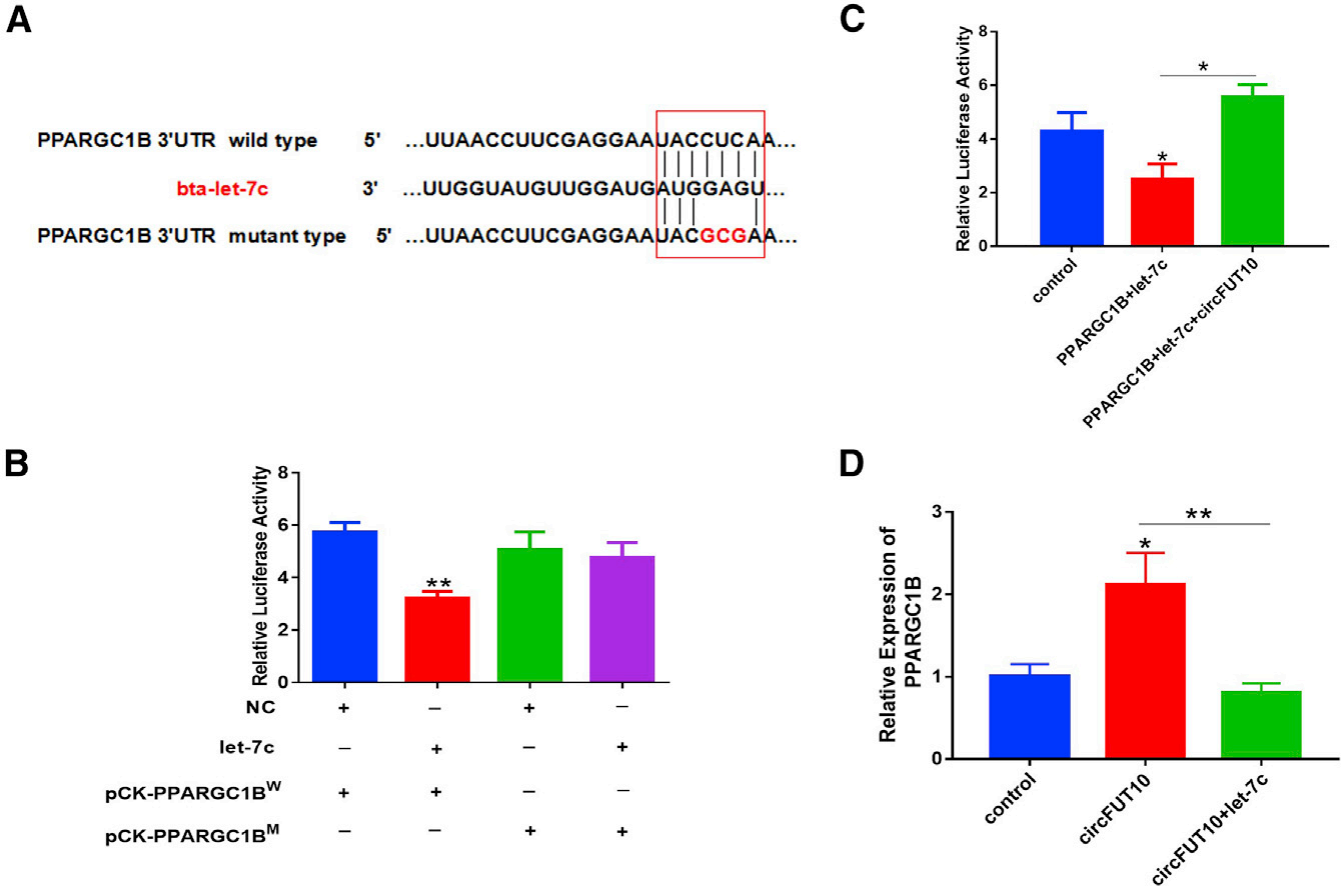
*PPARGC1B* Is a Target of let-7c (A) Predicted binding sites of let-7c in the 3′ UTR of *PPARGC1B*. The sequences indicated in red indicate certain mutated bases. (B) HEK293T cells were co-transfected with the wild-type or mutant *PPARGC1B* dual-luciferase vector and let-7c mimic, and the relative luciferase activity was analyzed 24 h after transfection. (C) The let-7c markedly inhibited *PPARGC1B* expression, and this effect was abrogated by *circFUT10* overexpression. (D) The *circFUT10* markedly promoted *PPARGC1B* expression, and this effect was abrogated by let-7c overexpression. ∗p < 0.05, ∗∗p < 0.01.

*PPARGC1B* was predicted by bioinformatics software TargetScan to be a potential target gene of let-7c. *PPARGC1B* has a potential binding site for let-7c in its 3′ UTR of seven bases (Figure 8A). Luciferase assay revealed that let-7c significantly decreased Rluc activity when co-transfected with let-7c mimic and pCK-*PPARGC1B*-3′ UTR-W (Figure 8B). Expression of *circFUT10* recovered the reduced Rluc activity induced by let-7c (Figure 8C). To confirm that *circFUT10* acts as a ceRNA to relieve the miRNA inhibitory effect on *PPARGC1B*, cattle adipocytes were treated with pCD2.1-*circFUT10* and/or the let-7c mimic. The results of qPCR showed that *circFUT10* markedly promoted *PPARGC1B* expression, and this effect was abrogated by let-7c overexpression (Figure 8D).

We measured the expression level of the *PPARGC1B* gene in calf and adult fat tissues and found higher expression of *PPARGC1B* in adult fat tissues compared to the level in calf fat tissues (Figure S5A). To determine the effect of *PPARGC1B* on bovine fat cells, *PPARGC1B* gene expression was knocked down with small interfering RNA (siRNA) (Figure S5B). The CCK-8 reagent was used to detect the proliferation status of adipocytes, and the results showed decreased signal after reducing *PPARGC1B* (Figure S5C). There were fewer positive EdU-stained cells after knockdown of *PPARGC1B* (Figure S5D). Furthermore, cell cycle was assayed by flow cytometry, and the results demonstrated a decrease in the proportion of S phase cells (Figures S5E and S5F). Finally, we found that the proliferation-related genes *PCNA* and *CDK2* were significantly reduced at the mRNA and protein levels (Figures S5G and S5H). After transfection with si-negative control (NC) or si-*PPARGC1B*, differentiation was induced for 6 days, and si-*PPARGC1B* promoted the expression of *PPARγ* and *C/EBPβ* at the mRNA and protein levels (Figures S6A and S6B). Oil red O staining showed that si-*PPARGC1B* significantly promoted the formation of lipid droplets (Figure S6C). Overall, all of these data demonstrated that *circFUT10* binding to let-7c inhibits bovine adipocyte cell differentiation by targeting *PPARGC1B* (Figure S7).

## Discussion

Of the ncRNAs, circRNAs are usually considered more stable due to the lack of 5′ to 3′ polarity and polyadenylated tail, but these RNAs may be abnormally regulated under certain disease conditions, such as colorectal cancer^28^ or Alzheimer’s disease.^29^ In addition, circRNA can have special functions, such as tissue-specific circRNAs involved in fetal development^30^ and accumulating with age in the brain of *Drosophila*.^31^ Taken together, these findings suggest that circRNAs are not by-products of transcriptional errors. It has been reported that circRNAs exhibit certain effects on bovine muscle^20^ and pig fat cells,^6^ but a function has not been reported for circRNAs on bovine fat cells. This is the first survey of the types and relative abundances of circRNAs in the fat tissue of calves and adult cattle.

circRNAs participate in the regulation of gene expression during skeletal muscle development.^6^ The localization of circRNAs in cells may correlate with different regulatory mechanisms. In the nucleus, circRNAs can be used as a molecular scaffold to recruit transcription factors to mediate gene transcription.^32^ In the cytoplasm, circRNAs can act as a molecular sponge for competitive binding to miRNAs, and relieve the inhibitory effects of miRNAs on target genes.^33^^,^^34^ For example, *circFGFR4* and *circFUT10* can regulate differentiation of cattle myoblasts via binding miR-107 and miR-133a to relieve their inhibition of *Wnt3a* and *SRF*, respectively. In this study, high-throughput RNA-seq analysis was performed to identify 8,809 and 9,937 circRNAs in calves and adult cattle fat tissues, respectively. The differential circRNAs of adult cattle and calves were screened and revealed that 151 circRNAs were downregulated significantly in adult cattle and 156 circRNAs were significantly upregulated relative to adult cattle in calves. We identified a circRNA, *circFUT10*, that is involved in fat tissue development. Functionally, *circFUT10* can act as a ceRNA by sponging let-7c to impede bovine adipocyte differentiation and promote cell proliferation.

circRNAs may function as miRNA sponges, and many studies have shown that circRNAs have biological effects by competitively binding miRNAs. For example, *circHIPK3* can promote angiotensin II-induced cardiac fibrosis by sponging miR-29b-3p,^35^ and *circ-FOXM1* facilitates cell progression by sponging miR-1304-5p in non-small-cell lung cancer,^34^ suggesting that the miRNA sponge effect of circRNAs is a vital mechanism. In this study, we identified *circFUT10* and observed high expression in fatty tissue. Using luciferase reporter assays, we found that *circFUT10* could sponge let-7c to relieve its inhibition on *PPARGC1B* and further inhibit adipocyte differentiation, while let-7c overexpression reversed this effect. The presence of let-7c promotes the expression of marker genes such as *PPAR-γ2* in human adipocytes.^16^ Consistent with these results, our experiments suggested that let-7c is a regulator of bovine fat cells. Therefore, we suspect that *circFUT10* indirectly promotes the transcription of *PPARGC1B* and then regulates cell proliferation and differentiation. Overall, these results demonstrated that *circFUT10* functions as a ceRNA to regulate cell proliferation and differentiation. While focusing on livestock breeding, we also learned that adipose tissue is not only an energy storage organ, but also an endocrine organ that can synthesize and secrete a variety of hormones and biologically active adipocytokines.^36^ Fat cells are closely related to physiological and pathological processes such as glucose and fat metabolism, obesity, diabetes, fatty liver, hyperlipidemia, and breast cancer.^37^ Because *circFUT10* acts to inhibit the differentiation of adipocytes, the potential association of *circFUT10* with obesity-related diseases, such as diabetes, should be explored further.

### Conclusions

In summary, our results provide the first comprehensive annotation of *circFUT10* in bovine adipogenesis and constitute a high-quality resource for future genomic studies and functional research. Thousands of circRNAs were annotated, several of which exhibit highly different abundances in calf and adult fatty tissues. We characterized and functionally explored an abundant circRNA—*circFUT10*. Our results reveal important roles of *circFUT10* in adipocyte proliferation and differentiation.

## Materials and Methods

### Sample Preparation

We collected six subcutaneous adipose samples of Qinchuan cattle from two developmental states each (three calf samples, 6 months after birth, and three adult samples, 24 months after birth) from a local slaughterhouse in Xi’an, P.R. China. Animal care and study protocols were approved by the Animal Care Commission of the College of Animal Science and Technology, Northwest A&F University.

### RNA-Seq Library Preparation and Sequencing Analysis

We subjected the RNA extract to electrophoresis on a denaturing agarose gel to assess total RNA yield and then quantified RNA concentrations using a NanoDrop spectrophotometer (NanoDrop Technologies, Wilmington, DE, USA) and an Agilent 2100 Bioanalyzer (Agilent, Santa Clara, CA, USA). Ribosomal RNA was depleted with a Ribo-Zero rRNA removal kit (Epicenter, Madison, WI, USA) before constructing RNA-seq libraries (to obtain sequence information of linear transcripts). Library preparation and Illumina sequencing analysis were described in a previous study.^38^ The raw sequencing dataset supporting the results of this study was deposited in the NCBI GEO database (https://www.ncbi.nlm.nih.gov/geo/). The data are accessible through GEO: GSE133735 (https://www.ncbi.nlm.nih.gov/geo/query/acc.cgi?acc = GSE133735).

### circRNA Identification and the Expression Levels Analysis

We used “find_circ” to identify circRNA. In this program, a 20-nt anchor sequence is extracted from both ends of the reads without alignment to the reference sequence and compared with each pair of anchor sequences again. If the 5′ end of the anchor sequence aligns to the reference sequence (starting and ending sites are denoted as A3 and A4, respectively), the 3′ end of the anchor sequence is aligned upstream of this site (starting and ending sites are denoted as A1 and A2, respectively), and there is a splice site (GT-AG) between A2 and A3 of the reference sequence; this read is then considered as a candidate circRNA. Finally, a candidate circRNA with a read count of two or more is considered as an identified circRNA. The expression levels of the circRNAs in each sample were counted, and the expression levels were normalized using TPM (transcripts per million), where TPM = (read count × 1,000,000)/libsize, where libsize (library size) is the sum of the sample circRNA read count.

### GO and Pathway Analysis

We used GO analysis (http://www.geneontology.org/) to characterize circRNA-hosting genes. GO terms provide information about the biological processes in which the genes are involved, either a cellular component or metabolic pathway, and highlight the related molecular function(s). We also performed KEGG (http://www.kegg.jp/) pathway analysis to obtain insight into the molecular interaction and reaction networks of circRNAs that were differentially expressed using DAVID (Database for Annotation, Visualization and Integrated Discovery, version 6.7; http://david.ncifcrf.gov/). In both cases, the −log_10_ p value denotes significant enrichment of a given GO term or pathway among upregulated and downregulated entities.

### Cell Culture

The adipose tissue of Qinchuan cattle was taken, and the primary cells of bovine fat were obtained by the tissue block culture method as previously described.^39^ HEK293T and bovine primary fat cells were cultured in Dulbecco’s modified Eagle’s medium (DMEM) supplemented with 10% fetal bovine serum (FBS), 100 μg/mL streptomycin, and 100 U/mL penicillin at 37°C with 5% CO_2_. Adipocytes at the stage of 80% confluence were plated at 5 × 10^5^ cells/well in 6-well plates or 1 × 10^4^ cells/well in 96-well plates (NEST, Wuxi, P.R. China). After growth to approximately 80% confluence, the cells were treated with let-7c mimic (2 μg/mL, RuiboBio, Guangzhou, China), pCD2.1-*circFUT10* (2 μg/mL), or let-7c mimic + pCD2.1-*circFUT10*. To induce differentiation of adipocytes, the culture medium was changed to high-glucose DMEM with 10% FBS.

### Plasmid Construction

The full length of *circFUT10* (Supplemental Information) was cloned into pCD2.1-ciR (Geneseed, Guangzhou, China), which contained a front and back circular frame, while the mock vector with no *circFUT10* sequence served as a control. To knock down *circFUT10*, siRNAs targeting the back-splice junction site of *circFUT10* and a siRNA-NC were synthesized by Ruibo (Guangzhou, China). We amplified the full length of *circFUT10* by psiCHECK2-*circFUT10*-F and psiCHECK2-*circFUT10*-R primers and inserted it into the psiCHECK-2 vector (Promega, Madison, WI, USA) at the 3′ end of the Renilla gene using restriction enzymes *Xho*I and *Not*I (Takara, Dalian, China) and T4 DNA ligase (pCK-*circFUT10*). Consistently, we generated the vectors of psiCHECK-*PPARGC1B*-W/Mut (pCK-PPARGC1B^W/M^). The genomic fragments, including let-7b, let-7c, let-7e, and let-7i, were cloned into pcDNA3.1vectors (Invitrogen, Carlsbad, CA, USA). The sequences of the primers used are presented in Table S3.

### RNA Preparation and Quantitative Real-Time PCR

Total RNA extraction, cDNA synthesis, and real-time qPCR were performed as previously described.^20^^,^^21^ For RNase R treatment, 1 μg of total RNA was incubated for 15 min at 37°C with 2 U/μg RNase R, and subsequently purified using an RNeasy MinElute cleaning kit (QIAGEN, Hilden, Germany). The qPCR analyses were performed using SYBR Green PCR master mix (Takara, Dalian, China). For each time point, qPCR was performed on three biological replicates. Gene expression levels were normalized to the housekeeping gene *β-actin*, and fold change was determined by the 2^−ΔΔCt^ method. The sequences of the primers used are presented in Table S4. We normalized the expression levels of *FUT10* in the control group to 1, and normalized the expression of *FUT10* in the overexpressed *circFUT10* group according to this standard.

### CCK-8 and EdU Assays

Adipocytes 24 h after transfection were investigated with the CCK-8 assay (Tiandz, Beijing, China) and Cell-Light EdU Apollo 567 *in vitro* imaging kit (RiboBio, Guangzhou, China), respectively. For detailed procedures, refer to the manufacturers’ instructions.

### Flow Cytometry

Twenty-four hours after transfection, cell cycle detection was performed by flow cytometry (FACSCalibur; Becton Dickinson, USA). Cell cycle was analyzed by using a cell cycle testing kit (Multisciences, Hangzhou, China). For detailed procedures, refer to the manufacturers’ instruction.

### Western Blot Analysis

Total proteins of bovine fat cells were extracted using radioimmunoprecipitation assay buffer (RIPA) buffer with 1% PMSF (Solarbio, Beijing, China) after transfection for 24 h or induction for 6 days after transfection. The protein concentration was determined by a bicinchoninic acid (BCA) kit (Beyotime, Shanghai, China), a 5× protein loading buffer (containing mercaptoethanol) was added to proteins at a ratio of 1:4, and then the sample was heated in boiling water for 3–5 min. Proteins were then separated by SDS-polyacrylamide gel electrophoresis and subsequently transferred to nitrocellulose membranes and blocked with milk powder solution for 2 h at room temperature. The membranes were then incubated overnight with the primary antibody of anti-*CDK2*, anti-*PCNA*, anti-*PPARγ*, anti-*C/EBPβ*, and anti-*β-actin* (Wanlei Bio, Shenyang, China). The membranes were then washed with PBS-Tween 20 and incubated for 1.5 h with horseradish peroxidase-conjugated secondary antibodies (Boster, Pleasanton, CA, USA). Finally, the membranes were imaged with an enhanced chemiluminescence (ECL) kit (Solarbio, Beijing, China) and quantified with the ImageJ program (Bio-Rad, USA).

### Oil Red O Staining

We induced the adipocytes for 6 days after transfection. The differentiated adipocytes were then washed twice with PBS and then fixed with 4% paraformaldehyde for 20 min. After washing twice with PBS, the cells were stained with oil Red O solution for 20 min and then imaged under the microscope. For quantification analysis, ImageJ software was used to perform statistical analysis according to the method described previously.^40^

### Luciferase Assays

HEK293T cells were cultured in DMEM with 10% FBS in 96-well plates, and the psi-check2-*circFUT10* plasmid was transfected together with the let-7c mimic. After 24 h, the Dual-Luciferase reporter (DLR) assay system kit (Promega, USA) was used to analyze firefly luciferase activity and Rluc activity according to the manufacturer’s instructions. The firefly luciferase activity served as a control to normalize the signal value.

### RIP Assay

A Magna RIP kit (Millipore, Bedford, MA, USA) was used to perform the RIP assay following the manufacturer’s protocol. Briefly, primary cattle fat cells were collected and lysed using RIP lysis buffer. The cell lysates were then incubated with magnetic beads conjugated with anti-Ago2 antibody (Abcam, Cambridge, UK). After the immunoprecipitated RNA was isolated, the abundance of *circFUT10* and let-7c in bound fractions was evaluated by qPCR analysis.

### Statistical Analyses

For each experiment we used three samples, each sample was tested three times, and the results were summarized in bar graphs using GraphPad Prism 7 software. The results are presented as mean ± SEM, and we performed a significant analysis using Student’s t test. p < 0.05 was considered statistically significant.

## Author Contributions

H.C., R.J. and H.L. designed the study. R.J., J.Y and C.S. performed the experiments and drafted the manuscript. Z.Y., R.J., X.W. and C.L. helped perform the experiments and analyzed the data. X.S., H.C., Y.H., and X.L. helped collect tissue samples.

## Conflicts of Interest

The authors declare no competing interests.

## Supplemental Information

Document S1. Figures S1–S7, Tables S2–S4, Supplemental Materials and Methods, and circFUT10

Table S1 The GO (Table S1-1) and KEGG (Table S1-2) Terms of Host Genes Encoding Differentially Expressed circRNAs

Document S2. Article plus Supplemental Information

## References

[1] Siersbæk R., Nielsen R., Mandrup S. (2012). Transcriptional networks and chromatin remodeling controlling adipogenesis. Trends Endocrinol. Metab..

[2] Algire C., Medrikova D., Herzig S. (2013). White and brown adipose stem cells: from signaling to clinical implications. Biochim. Biophys. Acta.

[3] Cui T.T., Xing T.Y., Chu Y.K., Li H., Wang N. (2017). Genetic and epigenetic regulation of PPARγ during adipogenesis. Yi Chuan.

[4] Farmer S.R. (2006). Transcriptional control of adipocyte formation. Cell Metab..

[5] Yan X., Zhu M.J., Dodson M.V., Du M. (2013). Developmental programming of fetal skeletal muscle and adipose tissue development. J Genomics.

[6] Li A., Huang W., Zhang X., Xie L., Miao X. (2018). Identification and characterization of circRNAs of two pig breeds as a new biomarker in metabolism-related diseases. Cell. Physiol. Biochem..

[7] Zhang H., Deng T., Ge S., Liu Y., Bai M., Zhu K., Fan Q., Li J., Ning T., Tian F. (2019). Exosome circRNA secreted from adipocytes promotes the growth of hepatocellular carcinoma by targeting deubiquitination-related USP7. Oncogene.

[8] Sanger H.L., Klotz G., Riesner D., Gross H.J., Kleinschmidt A.K. (1976). Viroids are single-stranded covalently closed circular RNA molecules existing as highly base-paired rod-like structures. Proc. Natl. Acad. Sci. USA.

[9] Hsu M.T., Coca-Prados M. (1979). Electron microscopic evidence for the circular form of RNA in the cytoplasm of eukaryotic cells. Nature.

[10] Danan M., Schwartz S., Edelheit S., Sorek R. (2012). Transcriptome-wide discovery of circular RNAs in Archaea. Nucleic Acids Res..

[11] Zhang C., Wu H., Wang Y., Zhao Y., Fang X., Chen C., Chen H. (2015). Expression patterns of circular RNAs from primary kinase transcripts in the mammary glands of lactating rats. J. Breast Cancer.

[12] Hansen T.B., Jensen T.I., Clausen B.H., Bramsen J.B., Finsen B., Damgaard C.K., Kjems J. (2013). Natural RNA circles function as efficient microRNA sponges. Nature.

[13] Kumar L., Shamsuzzama, Haque R., Baghel T., Nazir A. (2017). Circular RNAs: the emerging class of non-coding RNAs and their potential role in human neurodegenerative diseases. Mol. Neurobiol..

[14] Perriman R., Ares M. (1998). Circular mRNA can direct translation of extremely long repeating-sequence proteins in vivo. RNA.

[15] Xu P., Vernooy S.Y., Guo M., Hay B.A. (2003). The *Drosophila* microRNA mir-14 suppresses cell death and is required for normal fat metabolism. Curr. Biol..

[16] Esau C., Kang X., Peralta E., Hanson E., Marcusson E.G., Ravichandran L.V., Sun Y., Koo S., Perera R.J., Jain R. (2004). MicroRNA-143 regulates adipocyte differentiation. J. Biol. Chem..

[17] Zhang P., Du J., Wang L., Niu L., Zhao Y., Tang G., Jiang Y., Shuai S., Bai L., Li X. (2018). MicroRNA-143a-3p modulates preadipocyte proliferation and differentiation by targeting MAPK7. Biomed. Pharmacother..

[18] Guo X.Y., Sun F., Chen J.N., Wang Y.Q., Pan Q., Fan J.G. (2018). circRNA_0046366 inhibits hepatocellular steatosis by normalization of PPAR signaling. World J. Gastroenterol..

[19] Long T., Guo Z., Han L., Yuan X., Liu L., Jing W., Tian W., Zheng X.H., Tang W., Long J. (2018). Differential expression profiles of circular RNAs during osteogenic differentiation of mouse adipose-derived stromal cells. Calcif. Tissue Int..

[20] Wei X., Li H., Yang J., Hao D., Dong D., Huang Y., Lan X., Plath M., Lei C., Lin F. (2017). Circular RNA profiling reveals an abundant circLMO7 that regulates myoblasts differentiation and survival by sponging miR-378a-3p. Cell Death Dis..

[21] Li H., Yang J., Wei X., Song C., Dong D., Huang Y., Lan X., Plath M., Lei C., Ma Y. (2018). circFUT10 reduces proliferation and facilitates differentiation of myoblasts by sponging miR-133a. J. Cell. Physiol..

[22] Li H., Wei X., Yang J., Dong D., Hao D., Huang Y., Lan X., Plath M., Lei C., Ma Y. (2018). circFGFR4 promotes differentiation of myoblasts via binding miR-107 to relieve its inhibition of Wnt3a. Mol. Ther. Nucleic Acids.

[23] Sun J., Li M., Li Z., Xue J., Lan X., Zhang C., Lei C., Chen H. (2013). Identification and profiling of conserved and novel microRNAs from Chinese Qinchuan bovine longissimus thoracis. BMC Genomics.

[24] He H., Liu X. (2013). Characterization of transcriptional complexity during longissimus muscle development in bovines using high-throughput sequencing. PLoS ONE.

[25] Trapnell C., Williams B.A., Pertea G., Mortazavi A., Kwan G., van Baren M.J., Salzberg S.L., Wold B.J., Pachter L. (2010). Transcript assembly and quantification by RNA-seq reveals unannotated transcripts and isoform switching during cell differentiation. Nat. Biotechnol..

[26] Memczak S., Jens M., Elefsinioti A., Torti F., Krueger J., Rybak A., Maier L., Mackowiak S.D., Gregersen L.H., Munschauer M. (2013). Circular RNAs are a large class of animal RNAs with regulatory potency. Nature.

[27] Frost R.J., Olson E.N. (2011). Control of glucose homeostasis and insulin sensitivity by the Let-7 family of microRNAs. Proc. Natl. Acad. Sci. USA.

[28] Bachmayr-Heyda A., Reiner A.T., Auer K., Sukhbaatar N., Aust S., Bachleitner-Hofmann T., Mesteri I., Grunt T.W., Zeillinger R., Pils D. (2015). Correlation of circular RNA abundance with proliferation—exemplified with colorectal and ovarian cancer, idiopathic lung fibrosis, and normal human tissues. Sci. Rep..

[29] Lukiw W.J. (2013). Circular RNA (circRNA) in Alzheimer’s disease (AD). Front. Genet..

[30] Szabo L., Morey R., Palpant N.J., Wang P.L., Afari N., Jiang C., Parast M.M., Murry C.E., Laurent L.C., Salzman J. (2015). Statistically based splicing detection reveals neural enrichment and tissue-specific induction of circular RNA during human fetal development. Genome Biol..

[31] Westholm J.O., Miura P., Olson S., Shenker S., Joseph B., Sanfilippo P., Celniker S.E., Graveley B.R., Lai E.C. (2014). Genome-wide analysis of drosophila circular RNAs reveals their structural and sequence properties and age-dependent neural accumulation. Cell Rep..

[32] Li Z., Huang C., Bao C., Chen L., Lin M., Wang X., Zhong G., Yu B., Hu W., Dai L. (2015). Exon-intron circular RNAs regulate transcription in the nucleus. Nat. Struct. Mol. Biol..

[33] Kristensen L.S., Hansen T.B., Venø M.T., Kjems J. (2018). Circular RNAs in cancer: opportunities and challenges in the field. Oncogene.

[34] Liu G., Shi H., Deng L., Zheng H., Kong W., Wen X., Bi H. (2019). Circular RNA circ-FOXM1 facilitates cell progression as ceRNA to target PPDPF and MACC1 by sponging miR-1304-5p in non-small cell lung cancer. Biochem. Biophys. Res. Commun..

[35] Ni H., Li W., Zhuge Y., Xu S., Wang Y., Chen Y., Shen G., Wang F. (2019). Inhibition of circHIPK3 prevents angiotensin II-induced cardiac fibrosis by sponging miR-29b-3p. Int. J. Cardiol..

[36] Ghaben A.L., Scherer P.E. (2019). Adipogenesis and metabolic health. Nat. Rev. Mol. Cell Biol..

[37] Udler M.S., Kim J., von Grotthuss M., Bonàs-Guarch S., Cole J.B., Chiou J., Boehnke M., Laakso M., Atzmon G., Glaser B., Christopher D. Anderson on behalf of METASTROKE and the ISGC (2018). Type 2 diabetes genetic loci informed by multi-trait associations point to disease mechanisms and subtypes: A soft clustering analysis. PLoS Med..

[38] Li H., Wei X., Yang J., Dong D., Huang Y., Lan X., Plath M., Lei C., Qi X., Bai Y., Chen H. (2017). Developmental transcriptome profiling of bovine muscle tissue reveals an abundant GosB that regulates myoblast proliferation and apoptosis. Oncotarget.

[39] Li M., Sun X., Cai H., Sun Y., Plath M., Li C., Lan X., Lei C., Lin F., Bai Y., Chen H. (2016). Long non-coding RNA ADNCR suppresses adipogenic differentiation by targeting miR-204. Biochim. Biophys. Acta.

[40] Mehlem A., Hagberg C.E., Muhl L., Eriksson U., Falkevall A. (2013). Imaging of neutral lipids by oil red O for analyzing the metabolic status in health and disease. Nat. Protoc..

