# Supplementary material for: circRNA Profiling Reveals an Abundant circFUT10 that Promotes Adipocyte Proliferation and Inhibits Adipocyte Differentiation via Sponging let-7
Source: Mol Ther Nucleic Acids. 2020 Mar 30;20:491–501. doi: 10.1016/j.omtn.2020.03.011 (PMC7163053; doi:10.1016/j.omtn.2020.03.011)
Supplement: Document S1. Figures S1–S7, Tables S2–S4, Supplemental Materials and Methods, and circFUT10 [file mmc1.pdf]

OMTN, Volume *20*

Supplemental Information

circRNA Profiling Reveals an Abundant circFUT10 that Promotes Adipocyte Proliferation and Inhibits Adipocyte Differentiation via Sponging let-7

Rui Jiang, Hui Li, Jiameng Yang, Xuemei Shen, Chengchuang Song, Zhaoxin Yang, Xiaogang Wang, Yongzhen Huang, Xianyong Lan, Chuzhao Lei, and Hong Chen

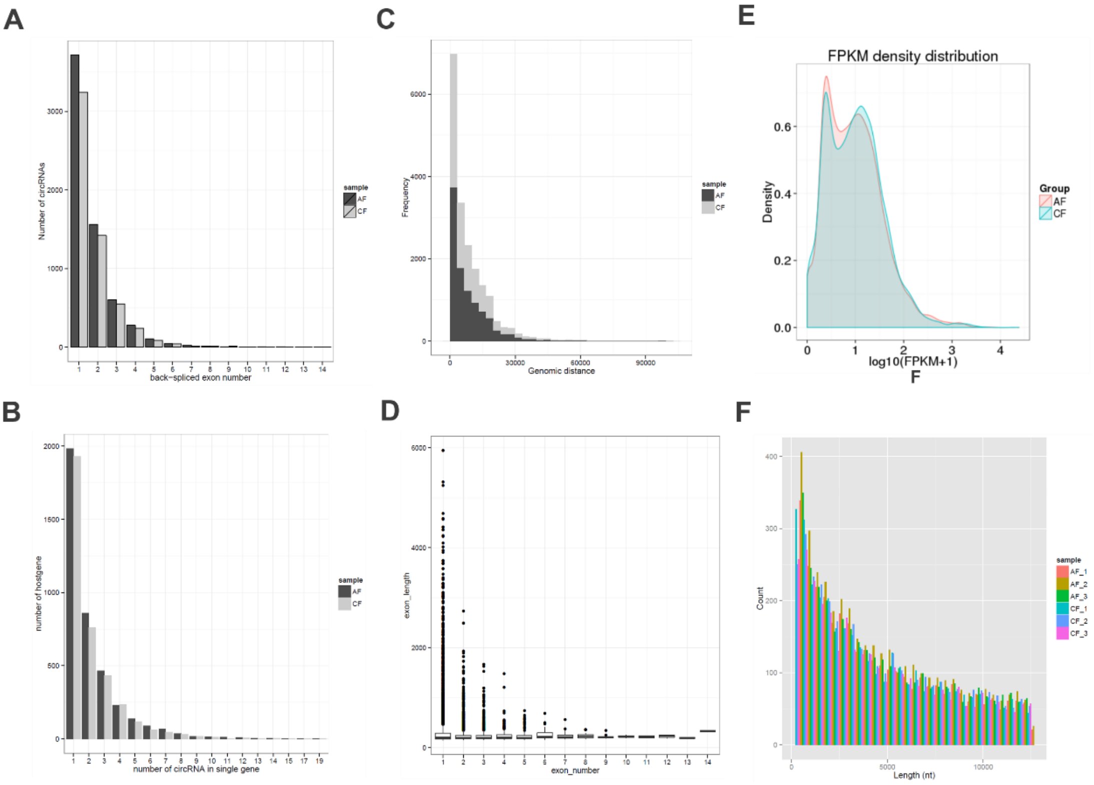
A
Number of circRNAs
back-spliced exon number
sample
AF
CF
C
Frequency
Genomic distance
E
FPKM density distribution
Density
log10(FPKM+1)
Group
AF
CF
B
number of hostgene
number of circRNA in single gene
D
exon_length
exon_number
F
Count
Length (nt)
AF_1
AF_2
AF_3
CF_1
CF_2
CF_3

Figure S1.

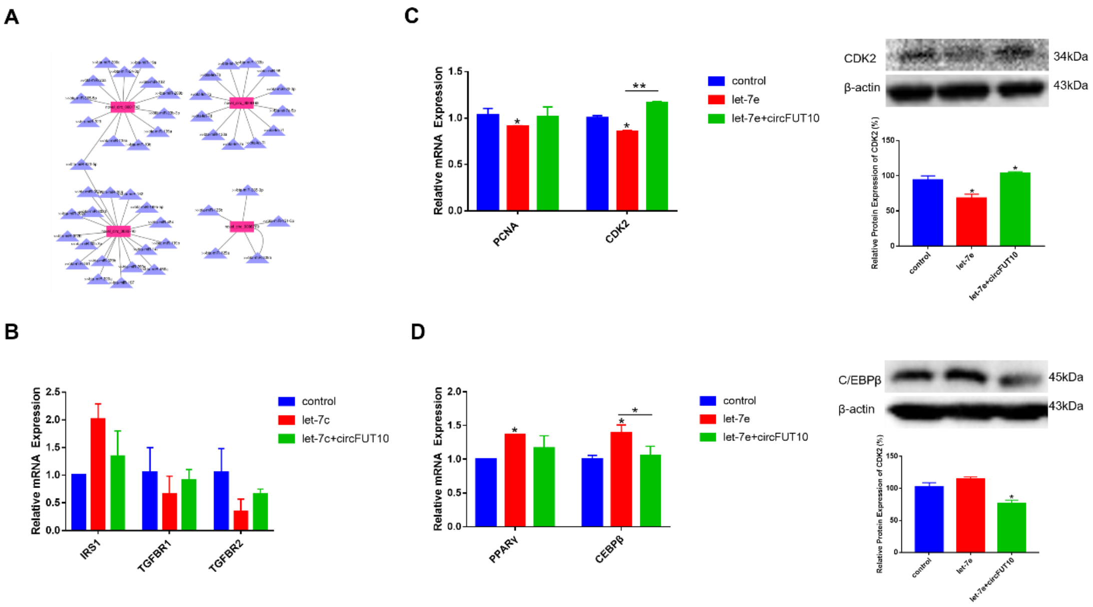
A
B
Relative mRNA Expression
2.5
2.0
1.5
1.0
0.5
0.0
IRS1
TGFBR1
TGFBR2
control
let-7c
let-7c+circFUT10
C
Relative mRNA Expression
1.5
1.0
0.5
0.0
PCNA
CDK2
*
*
**
control
let-7e
let-7e+circFUT10
CDK2
34kDa
β-actin
43kDa
Relative Protein Expression of CDK2 (%)
150
100
50
0
control
let-7e
let-7e+circFUT10
D
Relative mRNA Expression
2.0
1.5
1.0
0.5
0.0
PPARγ
CEBPβ
control
let-7e
let-7e+circFUT10
C/EBPβ
45kDa
β-actin
43kDa
Relative Protein Expression of CDK2 (%)
150
100
50
0
control
let-7e
let-7e+circFUT10

Figure S2.

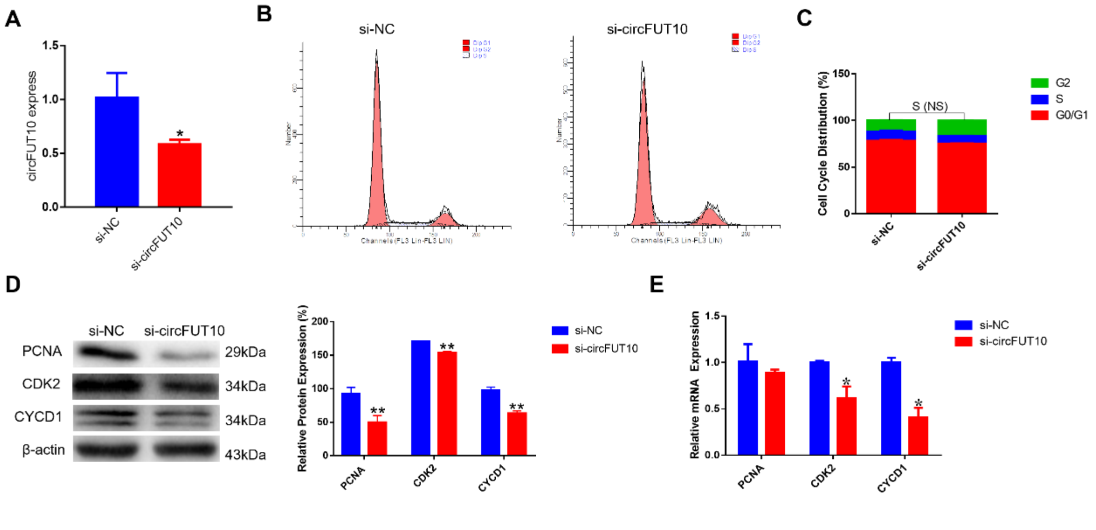

A
circFUT10 express
1.5
1.0
0.5
0.0
*
si-NC
si-circFUT10
B
si-NC
si-circFUT10
C
Cell Cycle Distribution (%)
150
100
50
0
S (NS)
G2
S
G0/G1
si-NC
si-circFUT10
D
si-NC
si-circFUT10
PCNA
CDK2
CYCD1
β-actin
29kDa
34kDa
34kDa
43kDa
Relative Protein Expression (%)
200
150
100
50
0
**
**
**
PCNA
CDK2
CYCD1
si-NC
si-circFUT10
E
Relative mRNA Expression
1.5
1.0
0.5
0.0
*
*
PCNA
CDK2
CYCD1
si-NC
si-circFUT10

Figure S3.

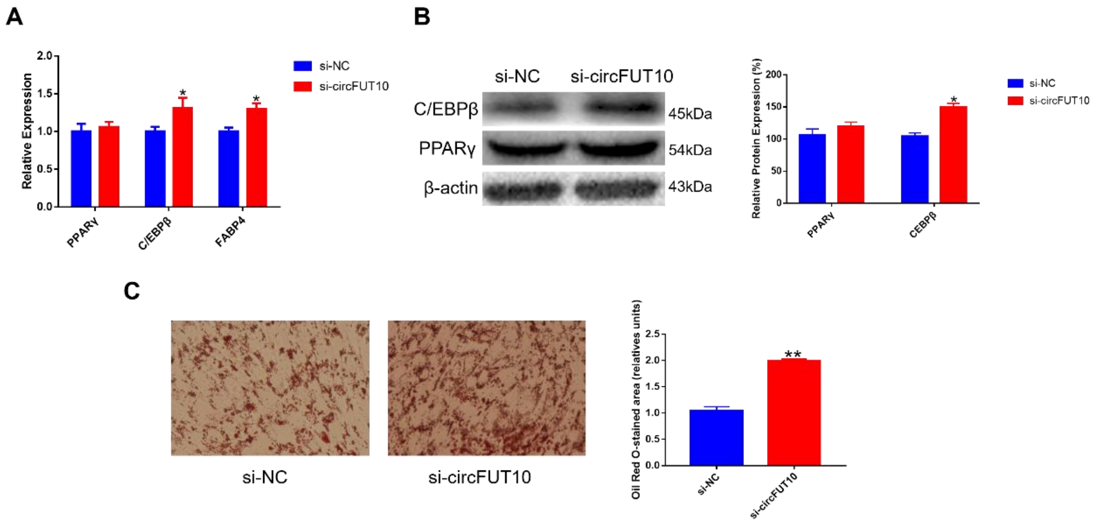
A
Relative Expression
2.0
1.5
1.0
0.5
0.0
PPARγ
C/EBPβ
FABP4
si-NC
si-circFUT10
B
si-NC
si-circFUT10
C/EBPβ
PPARγ
β-actin
45kDa
54kDa
43kDa
Relative Protein Expression (%)
200
150
100
50
0
PPARγ
CEBPβ
si-NC
si-circFUT10
C
si-NC
si-circFUT10
Oil Red O-stained area (relatives units)
2.5
2.0
1.5
1.0
0.5
0.0
si-NC
si-circFUT10

Figure S4.

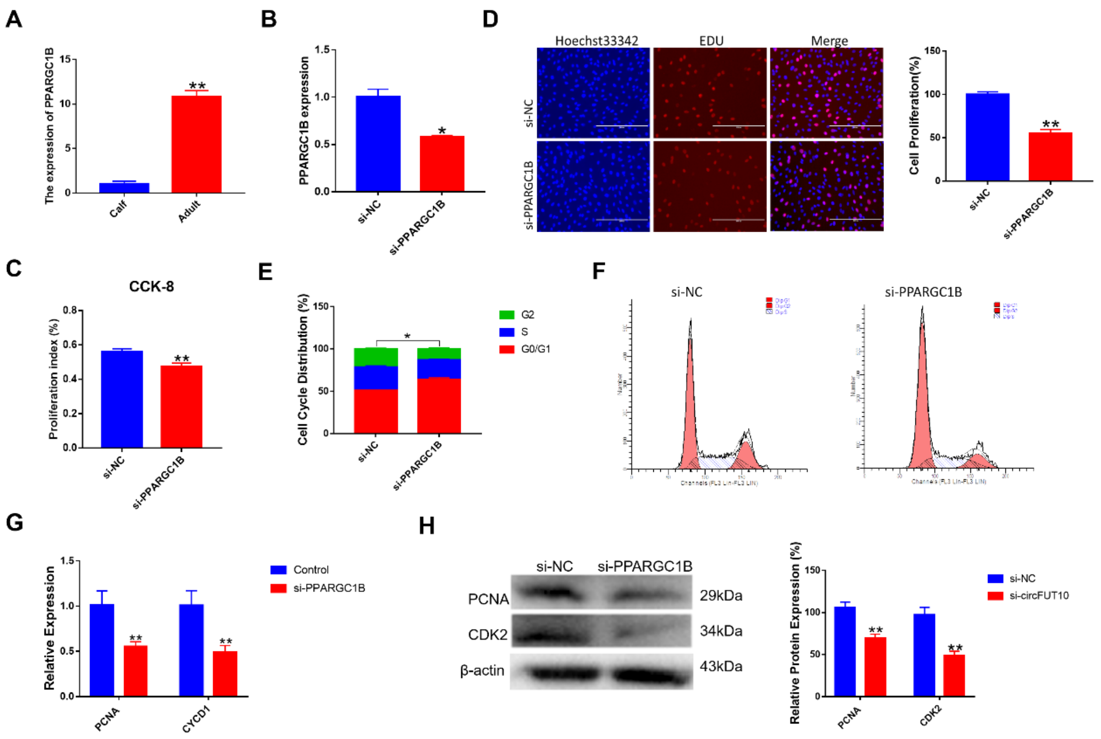
A
The expression of PPARGC1B
15
10
5
0
**
Calf
Adult
B
PPARGC1B expression
1.5
1.0
0.5
0.0
*
si-NC
si-PPARGC1B
D
Hoechst33342
EDU
Merge
si-NC
si-PPARGC1B
Cell Proliferation(%)
150
100
50
0
**
si-NC
si-PPARGC1B
C
CCK-8
Proliferation index (%)
0.8
0.6
0.4
0.2
0.0
**
si-NC
si-PPARGC1B
E
Cell Cycle Distribution (%)
150
100
50
0
*
G2
S
G0/G1
si-NC
si-PPARGC1B
F
si-NC
si-PPARGC1B
G
Relative Expression
1.5
1.0
0.5
0.0
**
**
Control
si-PPARGC1B
PCNA
CYCD1
H
si-NC
si-PPARGC1B
PCNA
29kDa
CDK2
34kDa
β-actin
43kDa
Relative Protein Expression (%)
150
100
50
0
**
**
si-NC
si-circFUT10
PCNA
CDK2

Figure S5.

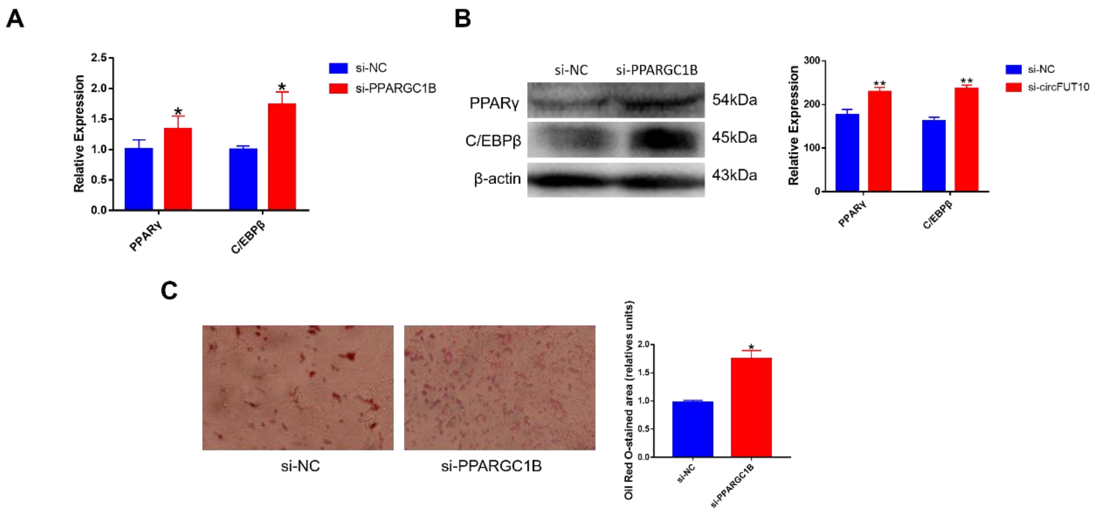
A
Relative Expression
2.5
2.0
1.5
1.0
0.5
0.0
si-NC
si-PPARGC1B
PPARγ
C/EBPβ
B
si-NC
si-PPARGC1B
PPARγ
C/EBPβ
β-actin
54kDa
45kDa
43kDa
Relative Expression
300
200
100
0
si-NC
si-circFUT10
PPARγ
C/EBPβ
C
si-NC
si-PPARGC1B
Oil Red O-stained area (relatives units)
2.0
1.5
1.0
0.5
0.0
si-NC
si-PPARGC1B

Figure S6.

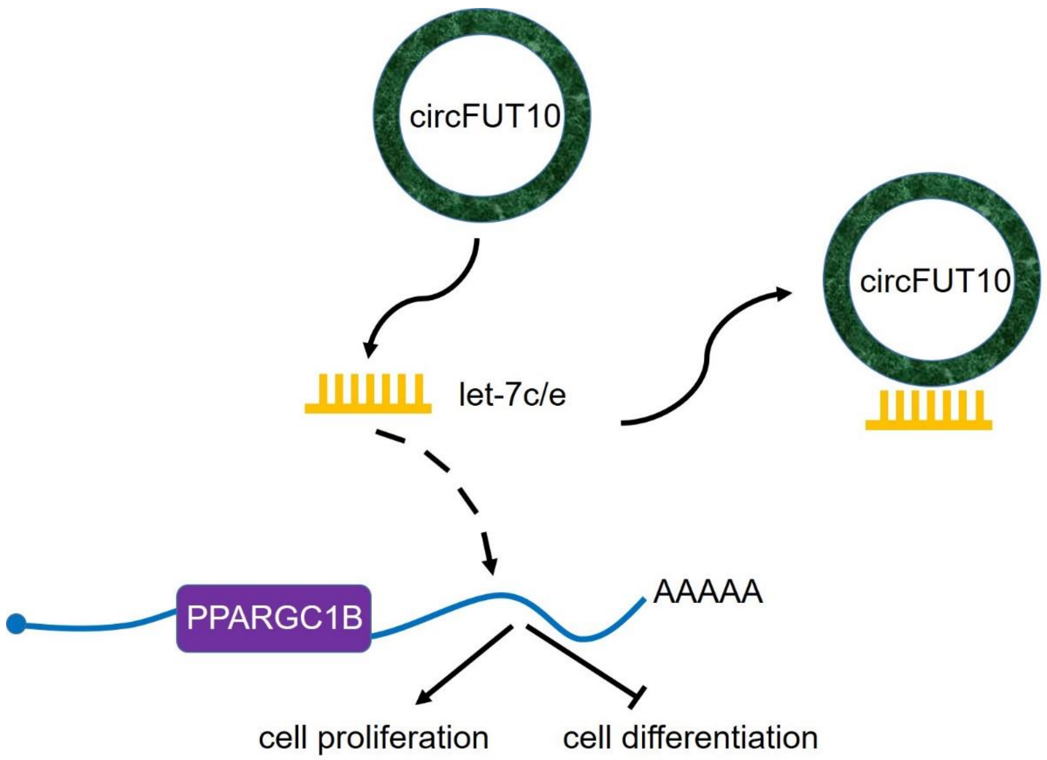
circFUT10
circFUT10
let-7c/e
PPARGC1B
AAAAA
cell proliferation
cell differentiation

Figure S7.

Figure S1. Characteristics of circular RNA in bovine fatty tissues. (A) The exon number of highly expressed circRNAs between adult period and calf fatty tissues; (B) Numbers of circRNAs produced by the same gene; (C) The genomic distance of the back-splicing site in most circRNAs is within 50 kb, with only a few circRNAs spanning >100 kb; (D-F) Exon length of circRNA.

Figure S2. (A) Competing endogenous RNA network in bovine fatty tissues. (B) The circFUT10 attenuates the effects of let-7c on IRS1, TGFBR1 and TGFBR2 genes. (C) The expression of proliferating cell nuclear antigen (PCNA) and cyclin-dependent-kinase 2 (CDK2) were detected by qPCR and Western blot. (D) The expression levels of PPARγ, C/EBPβ in bovine adipocyte were determined by qPCR and western blot. Quantitative analysis of western blot was performed using Image J. Values are mean ± SEM for three biological replicates; *P < 0.05; **P < 0.01

Figure S3. si-circFUT10 inhibits fat cells proliferation. (A) Interference efficiency of si-circFUT10. (B-C) Bovine fat cells were transfected with si-circFUT10 and cell phases were analyzed by flow cytometry. (D-E) The expression of CDK2, PCNA and CYCD1 were detected by Western blots and qPCR. The quantitative analysis of Western blot was performed using Image J. *$P < 0.05$; **$P < 0.01$.

Figure S4. si-circFUT10 promotes adipocyte differentiation. (A-B) Bovine fat cells were transfected with si-circFUT10 and the expression of PPARγ, C/EBPβ and FABP4 were detected by qPCR and Western blot. (C) Overexpression of si-circFUT10 promotes adipogenesis, as indicated by Oil Red O staining. The quantitative analysis of Western blot and Oil Red O were performed using Image J. *$P < 0.05$; **$P < 0.01$.

Figure S5. Effect of PPARGC1B gene on cell proliferation. (A) The expression of PPARGC1B gene in calf and adult fat tissues. (B) The efficiency of PPARGC1B knockdown was evaluated by qPCR. (C-D) si-PPARGC1B was transfected into bovine adipocytes for 24 hours, cell proliferation analysis using cell counting kit-8 (CKK-8) and EdU incorporation assays. The scale bars represent 100 mm. (E-F) Bovine primary fat cells were transfected with si-PPARGC1B, and cell phases were analyzed by flow cytometry. (G) The expression of proliferating cell nuclear antigen (PCNA) and cyclin D1 (CYCD1) were detected by qPCR. (H) The expression of proliferating cell nuclear antigen (PCNA) and cyclin-dependent-kinase 2 (CDK2) were detected by Western blot, quantitative analysis of Western blot was performed using Image J. Values are mean ± SEM for three biological replicates; *$P < 0.05$; **$P < 0.01$.

Figure S6. Effect of PPARGC1B gene on bovine adipocyte differentiation. (A-B) si-PPARGC1B was transfected into bovine adipocytes and induced to differentiate for six days. The expression of PPARγ, C/EBPβ were detected by qPCR and Western blot. (C) Oil Red O staining was detected in the group of si-NC and si-PPARGC1B, and quantitative analysis of Western blot and Oil Red O was performed using Image J. *$P < 0.05$; **$P < 0.01$.

Figure S7. Model of the action circFUT10 competitively sponging let-7 promotes adipocytes proliferation and inhibits adipocytes differentiation.

Table S2 The prediction of ΔG

name	let-7 family	sequence	ΔG (kcal/mol)
circFUT10	bta-let-7a	ugagguaguagguuguauaguu	-22.4
	bta-let-7b	ugagguaguagguugugugguu	-28.3
	bta-let-7c	ugagguaguagguuguaugguu	-28.9
	bta-let-7d	agagguaguagguugcauaguu	-22.6
	bta-let-7e	ugagguaggagguuguauagu	-25.9
	bta-let-7f	ugagguaguagauuguauaguu	-16.4
	bta-let-7g	ugagguaguaguuuguacaguu	-20.0
	bta-let-7i	ugagguaguaguuugugcuguu	-25.3

Table S3 primers for vector construction

**Name**	**Primer sequence 5’→3’**
pCD2.1-circFUT10-F	GGGGTACCGTCGTGGTTGAGCTGGGAA
Pcd2.1-circFUT10-R	GGGGATCCCATAGAAGAGGAAGGCTTTG
psiCHECK2-circFUT10-F	CCGCTCGAGGCACAAATGGAAGCAGAG
psiCHECK2-circFUT10-R	ATAAGAATGCGGCCGCGCTTTGGTCCTGTGATGA
psiCHECK2-PPARGC1B-Mut-F	CCGCTCGAGAAGTGCGGTAGTAGCTGAC
psiCHECK2-PPARGC1B-Mut-R	CCGCTCGAGGAATACGCGAATGAGCTAGAC
psiCHECK2-PPARGC1B-Wut-F	CCGCTCGAGAACAGCCTTAACCTTCGAGGA
psiCHECK2-PPARGC1B-Wut-R	ATAAGAATGCGGCCGCTGAACACAGCTGCACGTCTA
pcDNA3.1-let-7b-F	ATGGTACCGACGGCGGACCCTCTCAC
pcDNA3.1-let-7b-R	TAGGATCCACGTCACTGGGCCCCT
pcDNA3.1-let-7c-F	GGGGTACCATCCTTGCCAAGTCCTTAGGTG
pcDNA3.1-let-7c-R	CGGGATCCCTTCTTGCACAGAAATTGGCTCA
pcDNA3.1-let-7e-F	GGGGTACCCGAGAGACTGACTCCTGGGTT
pcDNA3.1-let-7e-R	CGGGATCCGGGGAGAAAGGAAAGGGACAAG
pcDNA3.1-let-7i-F	GGGGTACCGTCCTTAGGTGTATGGCTGC
pcDNA3.1-let-7i-R	CGGGATCCACAAGCGATCAGAAATACCATCTTG

Table S4 primers for qPCR

Gene	Primer pairs sequences (5′–3′)	Amplification length (bp)
GAPDH	F: TGAGGACCAGGTTGTCTCCTGCG	145
	R: CACCACCCTGTTGCTGTAGCCA	145
PPARγ	F: AGGATGGGGTCCTCATATCC	121
	R: GCGTTGAACTTCACAGCAAA	121
C/EBPα	F: TGGACAAGAACAGCAACGAG	130
	R: TTGTCACTGGTCAGCTCCAG	130
C/EBPβ	F: AAGATGCGCAACCTGGAGAC	124
	R: GCGTCTTGAACAAGTTCCGC	124
circFUT10	F: GCGGATGCTTGCTTCTTC	172
	R: GAGGCTCTGCTTCCATTTGT	172
FUT10	F: GGACATGCACAAATGGAAGCAG	197
	R: TAGGTCCGGTTGATGGTGAA	197
PCNA	F: TCCAGAACAAGAGTATAGC	94
	R: TACAACAGCATCTCCAAT	94
CDK2	F: TTTGCTGAGATGGTGACCCG	115
	R: TAACTCCTGGCCAAACCACC	115
cyclinD1	F: CCGTCCATGCGGAAGATC	108
	R: CAGGAAGCGGTCCAGGTAG	108
PPARGC1B	F: AGCTCTTCCAGATAATCGACAG	97
	R: GCAGCCAGACTCACAACATC	97
IRS1	F: TCAAGAGTGCCCACCTCAAC	185
	R: GGTCTTCATTCTGCTGTGATGTC	185
TGFBR1	F: GAGGCAACGCCATTACAGTG	166
	R: CAAATGGCCTGTCTCGTGGA	166
TGFBR2	F: CCCAAGTCGGTTAACAGCGA	85
	R: GCACGTCGCAGAACTTACAC	85

supplementary methods file

Genome reference and software used to assess linear species

The libraries we used were depleted of ribosomal RNA (rRNA−). The genome reference is NCBI_GCF_000003055.6; HT-Seq (v0.5.3p9) software was used to analyze the coverage of different known gene types of the species samples, using the model of union; We use different aligner for mRNA and circRNA. Quantitative analysis of mRNA was performed using cuffdiff[1] (http://cole-trapnell-lab.github.io/cufflinks/cuffdiff/index.html) software.

circFUT10

GTCGTGGTTGAGCTGGGAAAATTTGAAGGCAAGAAGTTTAAAAATTCTCATTTAAA

AGATGGACATGCACAAATGGAAGCAGAGCCTCTCCACCTCCATCCGTTCTTTAATA

GAGAAGGACTGACCCTGAACAGGAAGAAGACATTGGCAGCTGACAGCTTCCCCAT

CATGCTCTGGTGGTCCCCACTGACTGGGGAAACCGGAAGGCTCAGCCAGTGTGGG

GCGGATGCTTGCTTCTTCACCATCAACCGGACCTACCTCCATCATCACAGGACCAA

AGCCTTCCTCTTCTATG
